# *LungDiag*: Empowering artificial intelligence for respiratory diseases diagnosis based on electronic health records, a multicenter study

**DOI:** 10.1002/mco2.70043

**Published:** 2025-01-12

**Authors:** Hengrui Liang, Tao Yang, Zihao Liu, Wenhua Jian, Yilong Chen, Bingliang Li, Zeping Yan, Weiqiang Xu, Luming Chen, Yifan Qi, Zhiwei Wang, Yajing Liao, Peixuan Lin, Jiameng Li, Wei Wang, Li Li, Meijia Wang, YunHui Zhang, Lizong Deng, Taijiao Jiang, Jianxing He

**Affiliations:** ^1^ Department of Thoracic Surgery China State Key Laboratory of Respiratory Disease & National Clinical Research Center for Respiratory Disease the Key laboratory of Advanced Interdisciplinary Studies Center the First Affiliated Hospital of Guangzhou Medical University Guangzhou China; ^2^ Guangzhou National Laboratory Guangzhou China; ^3^ Guangzhou Women and Children's Medical Center Guangzhou China; ^4^ Department of Research and Developement Tianpeng Technology Co. Ltd Guangzhou China; ^5^ School of Health Policy and Management Nanjing Medical University Nanjing China; ^6^ Laboratory for Digital Intelligence & Health Governance Nanjing Medical University Nanjing China; ^7^ Department of Respiratory Disease The First People's Hospital of Kashi Prefecture Kashi China; ^8^ Department of Respiratory and Critical Care Medicine National Clinical Research Center of Respiratory Disease Key Laboratory of Pulmonary Diseases of Health Ministry Tongji Hospital Tongji Medical College Huazhong University of Science and Technology Wuhan Hubei China; ^9^ Department of Respiratory Disease The First People's Hospital of Yunnan Province Kunming China; ^10^ State Key Laboratory of Respiratory Disease The Key laboratory of Advanced Interdisciplinary Studies Center the First Affiliated Hospital of Guangzhou Medical University Guangzhou Guangdong China

**Keywords:** artificial intelligence (AI), electronic medical records (EHRs), natural language processing (NLP), respiratory diseases

## Abstract

Respiratory diseases pose a significant global health burden, with challenges in early and accurate diagnosis due to overlapping clinical symptoms, which often leads to misdiagnosis or delayed treatment. To address this issue, we developed *LungDiag*, an artificial intelligence (AI)‐based diagnostic system that utilizes natural language processing (NLP) to extract key clinical features from electronic health records (EHRs) for the accurate classification of respiratory diseases. This study employed a large cohort of 31,267 EHRs from multiple centers for model training and internal testing. Additionally, prospective real‐world validation was conducted using 1142 EHRs from three external centers. *LungDiag* demonstrated superior diagnostic performance, achieving an F1 score of 0.711 for top 1 diagnosis and 0.927 for top 3 diagnoses. In real‐world testing, *LungDiag* outperformed both human experts and ChatGPT 4.0, achieving an F1 score of 0.651 for top 1 diagnosis. The study emphasizes the potential of *LungDiag* as an effective tool to support physicians in diagnosing respiratory diseases more accurately and efficiently. Despite the promising results, further large‐scale multicenter validation with larger sample sizes is still needed to confirm its clinical utility and generalizability.

## Graphical abstract

*LungDiag* is an advanced system for diagnosing respiratory diseases, integrating deep learning‐based NLP to accurately extract clinical features at admission. It demonstrated high precision in identifying phenotypic entities and diagnosing respiratory diseases. By standardizing terminology and using detailed semantic modeling, *LungDiag* supports physicians in managing inpatient records and enhances clinical decision‐making amid diagnostic uncertainties.

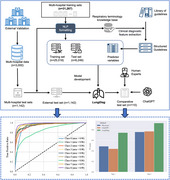

## 1 INTRODUCTION

Respiratory diseases, including chronic obstructive pulmonary disease (COPD), asthma, lung cancer, and pulmonary infections, are leading causes of morbidity and mortality worldwide, contributing significantly to the global health burden.^1^, ^2^, ^3^ Despite advances in treatment, the early diagnosis of these diseases remains a challenge due to the similarity of clinical symptoms, such as cough, dyspnea, and chest discomfort, across various respiratory conditions. Misdiagnosis or delayed diagnosis often leads to inappropriate treatment, poor patient outcomes, and increased healthcare costs.^4^

Accurate and timely diagnosis of respiratory diseases is essential for initiating appropriate treatments, improving patient outcomes, and reducing the burden on healthcare systems. Electronic health records (EHRs) contain valuable patient information, including clinical notes, diagnostic imaging, and laboratory results. These records integrate patient symptoms and clinical data to help formulate initial diagnoses upon admission. Nonetheless, it is noteworthy that these initial diagnoses may not consistently align with the ultimate discharge diagnoses, often exhibiting disparities, with rates as high as 49.2%.^5^, ^6^ To bridge this diagnostic gap, the imperative development of an intelligent diagnostic system for specific respiratory diseases, utilizing EHRs at admission, becomes evident.^7^

Artificial intelligence (AI), particularly natural language processing (NLP), has emerged as a transformative technology in healthcare, capable of analyzing unstructured data within EHRs to identify key clinical features. NLP allows for the extraction of symptoms, diagnoses, test results, and treatments, converting unstructured data into structured information that can be used for disease classification.^8^, ^9^ Several studies have demonstrated the potential of AI in improving diagnostic accuracy, but many existing models are focused on single diseases.^10^, ^11^ There remains a significant gap in the development of AI tools that can handle the complex and varied nature of respiratory diseases across multiple conditions.

To address this gap, we developed *LungDiag*, an AI‐based diagnostic system that leverages NLP to extract clinical features from EHRs and accurately classify a range of respiratory diseases. Our study utilized a large retrospective cohort for model training and testing. In addition, we compared the performance of *LungDiag* against both human experts and the *ChatGPT 4.0* AI model, with a focus on diagnostic accuracy and efficiency in real‐world clinical settings.

## 2 RESULT

### 2.1 Patient characteristics

**FIGURE 1:**
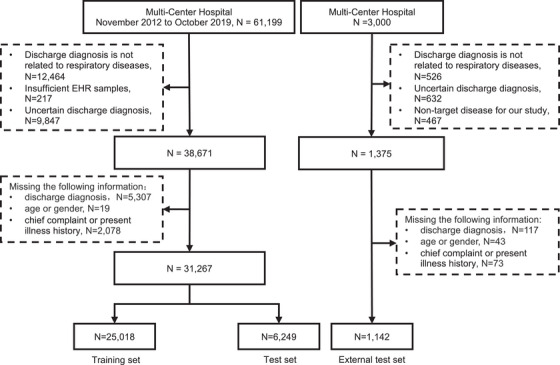
Flowchart for screening electronic health records meeting inclusion criteria.

**FIGURE 2:**
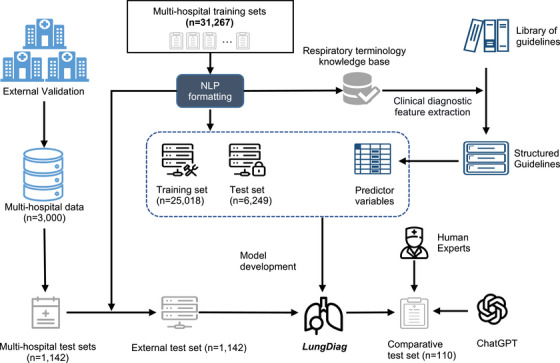
The design of the model and the usage of the datasets.

The study utilized 31,267 EHRs from the training centers, including 21,490 males and 9777 females (Figure 1). 80.7% of the diseases were respiratory diseases, with COPD being the most prevalent at 19.9%. The respiratory diseases EHRs were classified into 10 types, and the dataset was randomly divided into a training set (80%) and a test set (20%). An additional 1142 EHRs and 110 EHRs from three other centers were prospectively collected for external testing and expert‐AI comparison experiment (Table S1 and Figure 2).

### 2.2 Named entities recognition by the *LungDiag*

The recognition of named entities including disease names, symptoms, laboratory tests, image findings, medications, and surgical procedures provided the basis for identifying clinical features and ultimately classifying respiratory diseases. *LungDiag* performed satisfied in entity recognition, achieving an average precision of 0.883, recall of 0.918, and F1 score of 0.899 for all six types of entities in the test set (Table 1). This accuracy allowed for the annotation of a large number of clinical features from whole EHR database in the study, which were then used for disease classification. Additionally, we conducted phenotypic extraction testing on the dataset using ChatGPT 4.0, achieving precision, recall, and F1‐score values of 0.872, 0.775, and 0.821, respectively. These results indicated that *LungDiag* outperforms ChatGPT 4.0 in the task of phenotypic extraction.

**TABLE 1:** Performance of the natural language processing model for named entity recognition.

LungDiag	Precision	Recall	F1‐score
Disease names	0.876	0.886	0.881
Symptoms	0.921	0.956	0.938
Quantitative and qualitative tests	0.875	0.877	0.876
Image findings	0.814	0.930	0.868
Medications	0.821	0.736	0.776
Surgical procedures	0.780	0.623	0.692
Macro‐average precision	0.883	0.918	0.899

The Bi‐LSTM‐CRF deep learning model was utilized to perform named entity recognition on different categories of electronic health records.

### 2.3 Overall diagnostic performance of the *LungDiag*

**FIGURE 3:**
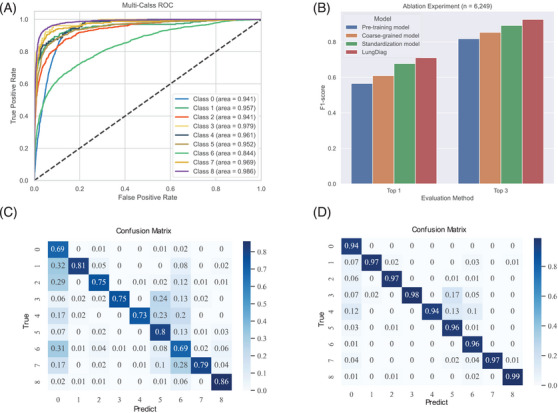
Performance evaluation of the *LungDiag*. (A) Receiver operating characteristic curves of *LungDiag* for different disease; (B) ablation experiment (the macro average F1‐score as the performance metric); (C) confusion matrix of the *LungDiag* for top 1 diagnosis; (D) confusion matrix of the *LungDiag* for top 3 diagnosis. Class 0: chronic obstructive pulmonary disease; Class 1: bronchial asthma; Class 2: bronchiectasis; Class 3: airway stenosis; Class 4: pulmonary hypertension; Class 5: lung space‐occupying lesions; Class 6: pulmonary infectious diseases; Class 7: pleural disease; Class 8: interstitial lung disease.

*LungDiag* demonstrated notable diagnostic accuracy, yielding top 1 diagnostic average precision, recall, and F1 score values of 0.763, 0.677, and 0.711, respectively (Table 2). Additionally, when it allowed to offer three answers, it achieved an average precision of 0.965, recall of 0.897, and an F1 score of 0.927 for top 3 diagnosis (Table 2). In total, the *LungDiag* had an average area under the curve of the receiver operating characteristic (AUROC) of 0.952 (95% CI 0.951–0.953) among all diseases in the test set (Figure 3A).

**TABLE 2:** Evaluation of *LungDiag*’s diagnostic performance for Top 1 and Top 3 differential diagnosis of respiratory diseases.

Disease	AUC	Top 1			Top 3		
		Precision	Recall	F1‐score	Precision	Recall	F1‐score
COPD	0.941 ± 0.001	0.693 ± 0.002	0.934 ± 0.005	0.796 ± 0.002	0.936 ± 0.002	0.995 ± 0.001	0.964 ± 0.001
Bronchial asthma	0.957 ± 0.002	0.812 ± 0.010	0.621 ± 0.003	0.704 ± 0.005	0.975 ± 0.007	0.879 ± 0.006	0.924 ± 0.004
Bronchiectasis	0.941 ± 0.002	0.754 ± 0.006	0.621 ± 0.011	0.681 ± 0.009	0.969 ± 0.005	0.918 ± 0.007	0.943 ± 0.005
Airway stenosis	0.979 ± 0.002	0.746 ± 0.012	0.610 ± 0.019	0.671 ± 0.010	0.983 ± 0.009	0.766 ± 0.017	0.861 ± 0.011
Pulmonary hypertension	0.961 ± 0.004	0.727 ± 0.009	0.545 ± 0.026	0.623 ± 0.019	0.941 ± 0.023	0.727 ± 0.018	0.821 ± 0.017
Lung space‐occupying lesions	0.952 ± 0.001	0.800 ± 0.005	0.747 ± 0.005	0.773 ± 0.005	0.963 ± 0.003	0.95 ± 0.002	0.956 ± 0.001
Pulmonary infectious diseases	0.844 ± 0.002	0.694 ± 0.005	0.563 ± 0.004	0.621 ± 0.003	0.960 ± 0.002	0.987 ± 0.002	0.973 ± 0.002
Pleural disease	0.969 ± 0.001	0.786 ± 0.009	0.563 ± 0.011	0.656 ± 0.009	0.971 ± 0.005	0.893 ± 0.011	0.93 ± 0.007
Interstitial lung disease	0.986 ± 0.001	0.857 ± 0.002	0.888 ± 0.002	0.872 ± 0.001	0.985 ± 0.003	0.963 ± 0.004	0.974 ± 0.003
Lung space‐occupying lesions	0.762 ± 0.003						
Lung cancer		0.922 ± 0.002	0.987 ± 0.002	0.953 ± 0.001			
Pulmonary tuberculosis		0.885 ± 0.013	0.534 ± 0.015	0.666 ± 0.011			

Abbreviation: AUC, area under the receiver operating characteristic curve.

Particularly, in the clinical setting, pulmonary nodules and masses are frequently detected as incidental findings on image finding, making the differentiation between lung cancer and tuberculosis a crucial diagnostic challenge. As such, a “two‐step” diagnostic approach is commonly employed, whereby both conditions are initially classified as “pulmonary occupying lesions,” followed by a thorough analysis of their distinctive characteristics to arrive at an accurate diagnosis. Finally, it demonstrated excellent diagnostic performance for lung cancer, achieving a precision, recall, and F1‐score of 0.919, 0.986, and 0.951, respectively (Table 2).

Significant differences were observed in the performance metrics among different diseases, as shown by the confusion matrix (Figure 3C,D). Specifically, in the evaluation of top 1 diagnosis, pulmonary infectious diseases were easily misclassified as other diseases, especially COPD. This may be because the pulmonary infectious diseases often coexist as a comorbidity in patient diagnosis.

Nevertheless, the robustness of our approach was evaluated through 10‐fold cross‐validation, demonstrating consistent overall performance across the different folds, indicating the reliability of our approach (Table S2).

### 2.4 Ablation experiment

We developed a deep learning‐based strategy as a baseline model for the diagnostic task. We then employed a coarse‐grained representation approach, followed by the use of standardized clinical features to construct machine learning prediction variables for disease prediction. Our results showed that the coarse‐grained representation used in the model outperformed the baseline model in diagnostic evaluations, with 4.4 and 3.6% improvement on top 1 and top 3 F1 scores, respectively (Table S3). Further, machine learning features constructed from clinical terminology standardization exhibited a certain degree of performance improvement in the final diagnosis evaluation. Specifically, the average F1 scores increased by 6.8 and 3.9% in the evaluations of top 1 and top 3, respectively (Table S3).

Furthermore, we transformed phenotype information from EHRs into fine‐grained phenotypes using the PIAT approach, resulting in the *LungDiag* model with better precision and effectiveness compared with the coarse‐grained model (Figure 3B). The fine‐grained phenotypic features exhibited superior diagnostic performance compared with the coarse‐grained features, with an average precision, recall, and F1 score increased by 2, 4, and 3.3% for top 1 diagnoses, and 2.3, 4, and 3.4% for top 3 diagnoses (Table S3). These findings suggest that the fine‐grained semantic information model can enable AI to learn more features.

### 2.5 Multicenter external test of the *LungDiag*

To assess the generalizability of the *LungDiag*, we used EHRs from three other hospitals for external test. The median age of the external dataset was 64 years old and the male‐to‐female ratio was 1.35:1, which was similar to the training set (Table S1). In the external test set, the average precision, recall, and F1‐score for the top 1 diagnosis was 0.637, 0.677, and 0.651, respectively. For the top 3 diagnoses, the model achieved an average precision, recall, and F1‐score of 0.973, 0.897, and 0.919, respectively (Table 3).

**TABLE 3:** Diagnostic performance of the *LungDiag* in multicenter external test.

	TOP 1			TOP 3		
Diseases	Precision	Recall	F1‐score	Precision	Recall	F1‐score
COPD	0.797	0.847	0.821	0.953	0.953	0.953
Bronchial asthma	0.721	0.772	0.746	0.981	0.930	0.955
Bronchiectasis	0.612	0.612	0.612	0.959	0.959	0.959
Airway stenosis	0.667	1.000	0.800	1.000	1.000	1.000
Pulmonary hypertension	0.412	0.333	0.368	1.000	0.381	0.552
Lung space‐occupying lesions	0.604	0.624	0.614	0.948	0.978	0.963
Pulmonary infectious diseases	0.722	0.664	0.692	0.952	0.987	0.969
Pleural disease	0.560	0.452	0.500	1.000	0.935	0.967
Interstitial lung disease	0.639	0.793	0.708	0.965	0.948	0.957

### 2.6 Comparison of performance of the *LungDiag* with that of human experts and ChatGPT 4.0

To further assess the diagnostic ability of the *LungDiag*, we compared its performance with that of human physicians and ChatGPT 4.0. We randomly selected 110 EHRs from independent cohort of the three centers and had nine certificated respiratory physicians manually diagnose the records (Table S4).

The *LungDiag* consistently outperformed all physicians, achieving F1 scores for top 1 and top 3 diagnoses at 0.745 and 0.927, respectively, while the highest top 1 and top 3 F1 scores achieved by physicians were 0.541 (*p* < 0.001, compared with *LungDiag*) and 0.834 (*p* < 0.001, compared with *LungDiag*), respectively (Table S4). When assisted by the *LungDiag*, the physicians would improve their top 1 and top 3 F1 scores to 0.834 (*p* < 0.001, compared with *physician only*) and 0.976 (*p* < 0.001, compared with *physician only*), respectively.

**FIGURE 4:**
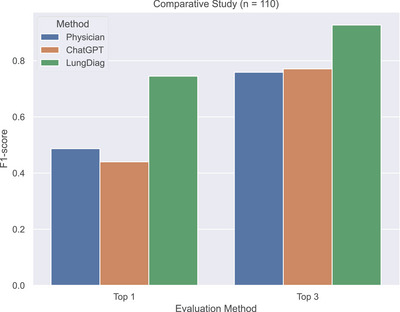
Comparison of diagnostic performance among *LungDiag*, human experts, and ChatGPT 4.0.

We also evaluated the diagnostic performance of ChatGPT 4.0 for top 1 and top 3 positions, achieving average F1‐scores of 0.440 (*p* < 0.001, compared with *LungDiag*) and 0.771 (*p* < 0.001, compared with *LungDiag*), respectively, which were comparable to those of human experts but still much lower than those of the *LungDiag* model (Table S5). Additionally, we applied specific embedding techniques to ChatGPT and conducted diagnostic inference testing, achieving average F1‐scores of 0.618 for top 1 and 0.818 for top 3 diagnostic performance. Compared with the initial performance without embedding, these results indicate an improvement; however, they remain inferior to the performance level of *LungDiag*. The results demonstrate the superior diagnostic ability of the *LungDiag* for respiratory diseases (Figure 4).

**FIGURE 5:**
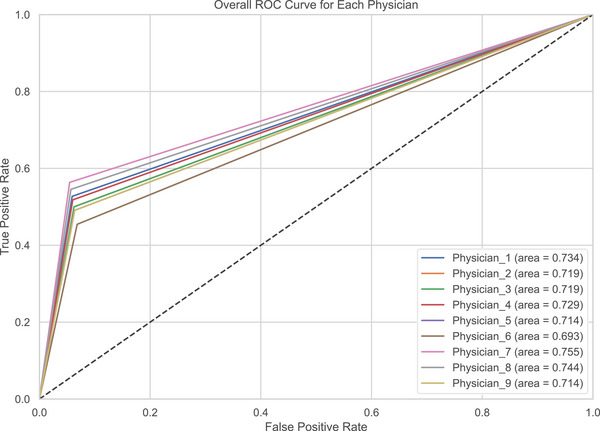
Receiver operating characteristic curves for each clinical expert.

To provide a more detailed comparison between the *LungDiag* model and clinical experts, we included the confusion matrix and ROC curves for the human expert diagnoses (Figures 5 and S1). The confusion matrix illustrates the distribution of correct and incorrect diagnoses made by the clinicians across different disease categories.

## 3 DISCUSSION

We developed an NLP approach, *LungDiag*, using deep learning to extract clinical information from EHRs and constructed an intelligent diagnostic system for 10 respiratory diseases, achieving satisfactory performance confirmed through external testing and comparative studies with human experts, as well as ChatGPT 4.0. To the best of our knowledge, this is the first study to predict multirespiratory diseases using unstructured EHR data based on NLP. Our self‐developed annotation platform and algorithms facilitates satisfactory annotation results with minimal human effort. Additionally, using fine‐grained standardized deep phenotyping effectively enhances the performance of machine learning models in disease diagnosis.

EHRs are crucial for healthcare provision, administration, and research. They contain structured and unstructured data, with structured data including diagnoses, prescriptions, and laboratory values, and unstructured data consisting of clinical notes and documentation.^12^ The adoption of EHRs has increased globally, turning them into sources of extensive healthcare data, also known as “big data.”^13^ Machine learning and deep learning techniques, are being used to analyze unstructured free‐text components in the medical domain. In this study, we highlight the potential of machine learning models in the healthcare setting, showcasing how a deep learning model, *LungDiag*, can provide robust and accurate diagnosis of respiratory diseases. This capability is critical given the high prevalence and complexity of respiratory diseases, which often require accurate and timely diagnosis for effective management. Importantly, the *LungDiag* model performed well not just in recognizing entities within EHRs, but also in extracting and interpreting clinical features, thereby assisting in disease classification. This performance underscores the crucial role of automated systems in navigating the complexity of EHRs. Such systems can streamline the identification of clinical features, which may otherwise be a time‐consuming task for medical professionals.

While the top 1 diagnostic average recall value of 0.677 indicates moderate sensitivity, this reflects the inherent challenges in diagnosing respiratory diseases based on EHRs. Many patients in our cohort presented with multiple coexisting respiratory conditions, leading to overlapping clinical features that make it difficult for the model to assign the correct primary diagnosis as the top choice. For instance, diseases like COPD, bronchiectasis, and pulmonary infections often occur together and share similar symptoms, complicating differentiation. Furthermore, the complexity and similarity of respiratory disease presentations can reduce the model's sensitivity for certain conditions, particularly when they are underrepresented in the training data. The lower recall for some diseases (<0.6) highlights the need for a more nuanced approach to distinguishing between diseases with overlapping symptoms. However, the substantial improvement in recall for the top 3 diagnoses (increasing to 0.897) suggests that *LungDiag* effectively includes the correct diagnosis within its top suggestions in most cases. This performance is clinically valuable, as it can aid physicians by narrowing down potential diagnoses and supporting decision‐making processes. In comparing the *LungDiag* with human physicians and another AI model, ChatGPT 4.0, we further offer valuable insights into the efficacy of AI in clinical settings. The *LungDiag* outperformed both human physicians and ChatGPT 4.0, suggesting a strong potential for AI‐assisted diagnosis. However, challenges remain in processing clinical notes and ensuring patient data privacy. NLP is utilized in clinical text mining, but further research is necessary to develop accurate and precise models for real‐world applications, particularly for diseases characterized by a complex range of symptoms, such as respiratory diseases.

Recent years have seen an increasing interest in the application of machine learning methodologies for the diagnosis and prediction of diseases, as evidenced by numerous studies in the field.^14^, ^15^, ^16^, ^17^ Traditional machine learning models, such as logistic regression,^18^ Naive Bayes,^19^ support vector machine,^20^ and random forest,^21^ have been widely employed in clinical studies. However, the LightGBM model offers distinct advantages over these traditional models. Notably, LightGBM exhibits superior training and prediction speeds, which is particularly beneficial when processing extensive medical datasets.^22^ Its ability to rapidly train high‐quality models and yield shorter prediction times satisfies the exigencies of real‐time performance requirements in the healthcare setting. Furthermore, the diagnosis of multiple respiratory diseases often leads to significant missing feature data in clinical EHRs due to the variability in patient presentations. However, LightGBM shows a remarkable capacity to handle such sparse feature data, thereby delivering superior accuracy. This capability, coupled with its efficient performance, positions LightGBM. Furthermore, the ablation experiment, which showed improved diagnostic performance with a fine‐grained representation, is a critical step in understanding how AI models could be optimized for diagnosing complex diseases. It also demonstrated how standardized clinical information can be effectively stratified to achieve the best results. The study should have delved further into how each granularity level affects the interpretability of results, which is a critical aspect in clinical settings.

In recent years, there has been a growing appreciation of the importance of leveraging real‐world clinical data, a resource that is often underutilized due to concerns surrounding data scarcity and confidentiality. For example, CheXbert, an innovative tool developed by researchers, utilizes BERT for the classification of free‐text radiological reports, outperforming traditional machine learning models that rely on feature engineering or human annotation.^23^ CheXbert achieves state‐of‐the‐art results in report labeling tasks on the MIMIC‐CXR dataset, which consists of a large collection of labeled chest radiographs, by learning from annotations and current rule‐based techniques. Despite these advancements, in the context of research involving Chinese EHRs, there is a conspicuous absence of relevant publicly available datasets. This deficiency considerably hampers progress in medical research. The current study addresses this issue by implementing meticulous data processing and standardization procedures, thereby ensuring the quality and consistency of the collected data. This multicenter dataset includes EHRs from more than 30,000 inpatients from the respiratory department, comprising comprehensive medical information. This information ranges from over 10 respiratory system‐related diseases to patient demographics (gender, age), chief complaints, history of present illness, radiology reports, and more. Furthermore, we have made a robust collection of 11,988 clinical terms related to respiratory system diseases openly available. This compilation incorporates ICD‐10 and ICD‐9CM‐3 codes, clinical guidelines relevant to respiratory system diseases, as well as clinical terminologies extracted from EHRs. The terms encompass disease names, symptoms, quantitative and qualitative tests, imaging findings, medications, and surgical procedures. Notably, a substantial portion of these terms has been translated into English to promote and facilitate future research endeavors. The provision of these resources is anticipated to significantly influence research in the field of Chinese EHRs.

The current algorithm primarily focuses on Chinese EHRs; however, we strongly believe that *LungDiag* can also be effectively applied other languages’ EHRs, especially for English. The field of medical information in English benefits from a wealth of high‐quality clinical terminology ontologies (such as UMLS, ICD) and well‐annotated data, providing us with abundant resources for the structuring and standardization of English EHRs. Furthermore, it is essential to highlight that our previously developed PIAT platform and algorithm have not only demonstrated effective deep phenotype annotation for Chinese medical records but also successfully supported deep phenotype recognition in English EHRs. Consequently, the processes of structuring, standardizing, and constructing fine‐grained semantic information models for both Chinese and English EHRs align seamlessly within *LungDiag* system. Taking these factors into consideration, we firmly believe that *LungDiag* is equally applicable to predictive diagnosis in English EHRs for respiratory system diseases.

This study presents an NLP‐based model for diagnosing respiratory diseases, but several limitations should be acknowledged. First, while the model was developed using a large retrospective cohort, the prospective validation was performed on a relatively small sample, limiting its robustness in real‐world settings. Larger multicenter studies are needed to further confirm the generalizability and clinical utility of *LungDiag*. Second, we used the BERT pretrained model for NLP tasks, but larger models like ChatGPT 4.0 may offer improved phenotype extraction performance.^24^ Third, although *LungDiag* performed well in diagnosing common respiratory diseases, it may struggle with rarer conditions or more complex cases that were underrepresented in the training data. Future studies should focus on addressing these limitations. Specifically, prospective validation with a larger dataset is necessary to assess the model's broader applicability. Additionally, exploring more advanced pretrained models, such as ChatGPT 4.0, may enhance diagnostic accuracy. Finally, despite our efforts to balance the dataset using Synthetic Minority Over‐sampling Technique (SMOTE) and to utilize macro‐averaged evaluation metrics, we acknowledge that class imbalance may still influence the model's performance, particularly for top 1 diagnosis. The overrepresentation of certain diseases could lead to higher apparent performance in predicting these conditions. We addressed this by conducting detailed analyses of the model's performance across all classes, and by discussing the challenges faced in diagnosing fewer common diseases. Future studies will focus on collecting more extensive datasets for underrepresented diseases and exploring advanced techniques to further mitigate the effects of class imbalance.

## 4 CONCLUSIONS

This multicenter study demonstrated the superior efficacy of *LungDiag* in accurately extracting clinical phenotypic entities with remarkable precision. Furthermore, *LungDiag* exhibited commendable diagnostic performance specifically for respiratory diseases. Importantly, the standardization of clinical medical terminology and the adoption of fine‐grained semantic information modeling have manifested potential in enhancing the intelligent diagnosis of diseases. The proposed approach in the study holds significant potential to aid physicians in handling voluminous inpatient records and to offer robust clinical decision support amidst diagnostic uncertainties.

## 5 METHODS

### 5.1 Study design and dataset

This study obtained approval from the Ethics Committee of the training centers: National Center for Respiratory Medicine at *Liwan*, The First Affiliated Hospital of Guangzhou Medical University at *Yuexiu* (ES‐2022‐70), as well as from three additional external testing medical centers: the First People's Hospital of Yunnan Province (ES‐2024‐069‐02), the First People's Hospital of Kashgar (ES‐2024‐158‐02), and Tongji Hospital of Tongji Medical College of Huazhong University of Science and Technology (ES‐2024‐K135‐01). Due to the noninterventional nature of the study, informed consent was waived.

EHRs of in‐hospital patients with respiratory disease at the training centers between November 1, 2012 and October 30, 2019 were retrospectively gathered for model training and internal testing. EHRs of patients from the other three hospitals were prospectively collected between June 1, 2022 and September 30, 2022 in real world for external testing. The *LungDiag* system encompasses two primary functions: (1) identification of distinct clinical phenotypes/attributes from EHRs using deep learning‐based NLP and (2) respiratory disease classification based on the recognized fine‐grained clinical phenotypes employing machine learning. We ensured that all patient data were fully anonymized prior to extraction from the hospital's EHR system. This process involved the complete deidentification of sensitive information, with only coded data being used for analysis. Access to this data was restricted to authorized research personnel, who were the only individuals capable of linking the codes back to patient records if necessary for study purposes.

### 5.2 Data selection and preprocessing

We selected patients admitted to the respiratory department, ensuring that the primary diagnosis was related to respiratory diseases. As a result, we used the first diagnosis as the gold standard for building and validating the *LungDiag* model, rather than including all discharge diagnoses. This approach allowed for a more focused and accurate classification of respiratory conditions. We selected the first admission record for patients with multiple admissions and obtained all discharged cases in the training sets. Consecutive EHRs included patient information, chief complaint, medical history, physical examination, imaging findings, blood tests, and discharge diagnosis, with the latter serving as the gold standard. We excluded EHRs lacking necessary information, those with unrelated discharge diagnoses, and some respiratory diseases with insufficient cases.

The discharge diagnoses were standardized according to International Classification of Diseases‐10 (ICD‐10), resulting in 10 identified disease types: COPD, bronchial asthma, bronchiectasis, pulmonary infectious diseases, airway stenosis, pulmonary hypertension (including pulmonary heart disease), lung cancer, pulmonary tuberculosis, pleural disease, and interstitial lung disease. The training datasets was randomly divided into training and internal testing sets at an 8:2 ratio, with the patient screening process and data utilization and model design illustrated in Figures 1 and 2, respectively.

### 5.3 Construction of NLP model

In order to accurately predict respiratory diseases using EHRs, it is essential to extract and standardize clinical features and phenotypic attributes. To accomplish this, we developed an NLP algorithm that involved manual annotation, deep learning techniques, and standardization of clinical features and clinical phenotype terms in EHRs.

We used a Bi‐LSTM‐CRF model^25^ for named entity recognition, with a “BIO” tagging schema^26^ to label sequences of phenotypic entities such as disease names, symptoms, quantitative and qualitative tests, image findings, medications, and surgical procedures (Figure S2). The hyperparameters and values for the models are presented in Table S6. We also employed an interactive intelligent annotation system, PIAT,^27^ to annotate clinical data from EHRs. A total of 1225 EHRs were manually annotated by physicians to train and test the model.

To further refine the phenotypic features extracted by the deep learning model, we screened for respiratory disease‐related features that were exclusively selected from clinical guidelines and authoritative medical textbooks pertaining to the diseases of interest. A respiratory terminology knowledge base was utilized to extract features from these guidelines and medical textbooks, which were reviewed and corrected by two clinicians. Ultimately, a total of 442 clinical features related to respiratory disease diagnosis were extracted and standardized into 252 clinical features using UMLS.^28^, ^29^

In addition to standardizing clinical features, it is also important to establish associations between phenotypes and attributes (Figure S3). We employed an association rule to establish such associations after their recognition, resulting in 10 phenotypic attributes for deep phenotyping study aimed at the intelligent diagnosis of respiratory diseases (Table S7).

The NLP algorithm, annotation system, and standardization techniques provide a robust foundation for accurately predicting respiratory diseases using EHRs.

### 5.4 Construction of the classification model

In this study, we focused on developing accurate diagnostic models for respiratory diseases, which can be challenging due to imbalanced class sizes and the complexity of clinical data. We addressed the issue of class imbalance inherent in our dataset by employing the SMOTE.^30^ SMOTE was used to generate synthetic samples for minority classes during the training phase, effectively balancing the class distribution and reducing bias toward majority classes. This approach allowed the model to learn equally from all classes, enhancing its ability to accurately predict both common and rare respiratory diseases.

We then applied BERT^31^ and LightGBM,^32^ for the classification task. BERT is a state‐of‐the‐art deep learning method for NLP, which was used to encode EHR texts and predict the diagnosis of respiratory diseases. LightGBM is a gradient boosting framework that is well suited for handling large‐scale datasets with many features, which was used to predict respiratory diseases based on clinical feature variables. Table S6 shows the details of the hyperparameters.

In addition, we investigated the impact of standardizing clinical terms on disease diagnosis and prediction. We also applied a fine‐grained semantic information model to standardize clinical phenotypes and attributes.

### 5.5 Comparison the performance of the *LungDiag* with human experts and ChatGPT 4.0

We conducted a comparative study to evaluate the diagnostic performance of *LungDiag* in comparison with human experts. An independent cohort of 110 respiratory disease patient records from the three other external centers was prospectively collected in real world. Nine respiratory clinical physicians with qualification certificates manually diagnosed the 110 records by selecting the three options with the highest probability from 10 disease options provided for each case. Initially, physicians were asked to independently review the medical records and provide their top three diagnoses. Once their initial diagnoses were recorded, they were presented with the *LungDiag* diagnosis and given the chance to revise their diagnoses based on the AI's input. This second set of diagnoses, which factored in the *LungDiag* recommendations, was treated as the physician‐assisted diagnosis. The diagnostic performance of each physician was evaluated in the top 1 and top 3 diagnosis categories using precision, recall, and F1 score metrics.

We also compared *the LungDiag* with ChatGPT 4.0 for respiratory diseases classification using the same dataset of 110 records. ChatGPT 4.0 is a highly efficient AI model developed by OpenAI that has demonstrated success in natural language tasks. We evaluated ChatGPT 4.0′s diagnostic performance by inputting the EHR text and providing 10 disease options for it to choose from. We utilized ChatGPT 4.0 to classify respiratory diseases using the original Chinese text without translation into English. Finally, we evaluated ChatGPT 4.0′s diagnostic performance in top 1 and top 3 for different disease categories.

### 5.6 Statistical analysis

We assessed the performance of the *LungDiag* using precision, recall, and F1 score metrics in NLP tasks. Specifically, we evaluated the top 1 and top 3 precision, recall, and F1 score in disease diagnoses. To evaluate the stability and robustness of the *LungDiag* model, we performed repeated experiments using 10‐fold cross‐validation on the training dataset. The dataset was randomly partitioned into 10 equal subsets. In each iteration, one subset was used as the validation set, while the remaining nine subsets were used for training. This process was repeated 10 times, ensuring that each subset was used as the validation set once. Performance metrics such as precision, recall, F1‐score, and AUC were calculated for each fold. The final results are reported as mean ± standard deviation across all folds to provide a comprehensive assessment of the model's stability. To estimate the 95% confidence intervals for the AUROC, we employed the bootstrapping method. Specifically, we performed 1000 bootstrap resamples of the test dataset and recalculated the AUROC for each resample to generate a distribution of AUROC values. The standard error was calculated from this distribution using the stats module from the SciPy library. The 95% confidence intervals were then determined by taking the 2.5th and 97.5th percentiles of the bootstrapped AUROC distribution. This nonparametric approach allows for a robust estimation of the confidence intervals without assuming a specific distribution of the AUROC estimates.

We employed the Wilcoxon signed‐rank test using the Wilcoxon function from SciPy to statistically compare the diagnostic performance of the *LungDiag* system against that of clinicians and ChatGPT 4.0. The resulting *U* statistic and *p* values provided insights into whether these differences were statistically significant, particularly in the diagnosis of specific respiratory diseases. We used *Python version 3.8* for all statistical analyses and Matplotlib for generating figures.

## AUTHOR CONTRIBUTIONS

Hengrui Liang, Tao Yang, Lizong Deng, Taijiao Jiang, and Jianxing He gave conception and design of this study. Lizong Deng, Taijiao Jiang, and Jianxing He offered the administrative support. Hengrui Liang, Tao Yang, Yilong Chen, Bingliang Li, and Zeping Yan developed the *LungDiag* system. Zihao Liu and Wenhua Jian assisted to improve the system. Hengrui Liang, Tao Yang, Yilong Chen, Bingliang Li, and Zeping Yan conducted experimental analysis. Weiqiang Xu, Luming Chen, Yifan Qi, Zhiwei Wang, Yajing Liao, Peixuan Lin, Jiameng Li, Wei Wang, Li Li, Meijia Wang, and YunHui Zhang leaded the data collection. All the other authors participated in the data collection. The manuscript was written by Hengrui Liang and Tao Yang. All authors read and approved the manuscript. Hengrui Liang and Tao Yang have accessed and verified the data. Hengrui Liang, Tao Yang, and Jianxing He were responsible for the decision to submit the manuscript. All authors have read and approved the final manuscript.

## CONFLICT OF INTEREST STATEMENT

Author Yilong Chen is an employee of Tianpeng Technology Co. Ltd., but has no potential relevant financial or nonfinancial interests to disclose. The other authors have no conflict of interest to declare. The study sponsor was responsible for the collection, analysis, and interpretation of the data, for the writing of the manuscript, and for the decision to submit the manuscript for publication.

## ETHICS STATEMENT

This study obtained approval from the Ethics Committee of the training centers: National Center for Respiratory Medicine at *Liwan*, The First Affiliated Hospital of Guangzhou Medical University at *Yuexiu* (ES‐2022‐70), as well as from three additional external testing medical centers: the First People's Hospital of Yunnan Province (ES‐2024‐069‐02), the First People's Hospital of Kashgar (ES‐2024‐158‐02), and Tongji Hospital of Tongji Medical College of Huazhong University of Science and Technology (ES‐2024‐K135‐01). Due to the noninterventional nature of the study, informed consent was waived.

## Supporting information



Supporting information

Supporting information

Supporting information

## Data Availability

The full raw image data recorded in the segmentation and phase classification tasks during the current study will be released at publication (contact hengrui_liang@163.com for the raw data). The raw code for this study has been uploaded to GitHub and can be accessed via the following link: [https://github.com/yangtao1025/LungDiag]. Access to the code is controlled and requires approval from the corresponding author.
