# Supplementary material for: LungDiag: Empowering artificial intelligence for respiratory diseases diagnosis based on electronic health records, a multicenter study
Source: MedComm (2020). 2025 Jan 12;6(1):e70043. doi: 10.1002/mco2.70043 (PMC11725045; doi:10.1002/mco2.70043)
Supplement: Supplementary file 1 — Supporting information [file MCO2-6-e70043-s001.docx]

**Supplementary Materials**

**Supplement tables legends:**

**Table S1:** Patient characteristics in training and test set

**Table S2:** Diagnostic performance of the ***LungDiag*** classifier for respiratory diseases using 10-fold cross-validation

**Table S3:** Performance of ablation experiments for each respiratory system diseases

**Table S4:** Diagnostic performance for respiratory diseases of physicians, ***ChatGPT 4.0*** and the ***LungDiag***

**Table S5:** Comparison of diagnostic performance of physicians, ***ChatGPT 4.0*** and the ***LungDiag***

**Table S6:** Hyperparameters of the ***LungDiag*** model

**Table S7:** Definition and typical values of the attribute

**Supplement figure legends:**

**Figure S1:** Confusion matrix of the expert for top 3 and top 1 diagnosis.

**Figure S2: Algorithm structure diagram**

**Figure S3:** Phenotype and attribute correlation algorithm

**Table S1:** Patient characteristics in training and test set

| Characteristic | Training set | Internal test set | Total | External test set |
| --- | --- | --- | --- | --- |
| Age (years) | 61.61 (61.41-61.80) | 65.80 (65.45-66.16) | 62.44 (62.27-62.61) | 61.41 (60.52-62.30) |
| Gender |  |  |  |  |
| Male | 17,082 (68.28%) | 4,408 (70.54%) | 21,490 (68.73%) | 658 (57.62%) |
| Female | 7,936 (31.72%) | 1,841 (29.46%) | 9,777 (31.27%) | 484 (42.38%) |
| Diseases |  |  |  |  |
| COPD | 6,795 | 1,700 | 8,495 | 385 |
| Bronchial asthma | 1,057 | 264 | 1,321 | 57 |
| Bronchiectasis | 2,436 | 609 | 3,054 | 49 |
| Pulmonary infectious diseases | 5,841 | 1,459 | 7,300 | 446 |
| Airway stenosis | 309 | 77 | 386 | 2 |
| Pulmonary hypertension | 358 | 88 | 446 | 21 |
| Lung cancer | 3,153 | 813 | 3,966 | 67 |
| Pulmonary tuberculosis | 694 | 147 | 841 | 26 |
| Pleural disease | 1,043 | 261 | 1,304 | 31 |
| Interstitial lung disease | 3,332 | 831 | 4,163 | 58 |
| Total | 25,018 | 6,249 | 31,267 | 1,142 |

COPD, chronic obstructive pulmonary diseases

**Table S2:** Diagnostic performance of the ***LungDiag*** classifier for respiratory diseases using 10-fold cross-validation

| **Fold_num** | **Precision** | **Recall** | **F1-score** |
| --- | --- | --- | --- |
| 0_Fold | 0.657 | 0.633 | 0.645 |
| 1_Fold | 0.741 | 0.666 | 0.702 |
| 2_Fold | 0.716 | 0.622 | 0.665 |
| 3_Fold | 0.762 | 0.661 | 0.708 |
| 4_Fold | 0.684 | 0.613 | 0.646 |
| 5_Fold | 0.655 | 0.564 | 0.606 |
| 6_Fold | 0.756 | 0.648 | 0.697 |
| 7_Fold | 0.791 | 0.703 | 0.745 |
| 8_Fold | 0.779 | 0.719 | 0.748 |
| 9_Fold | 0.701 | 0.624 | 0.660 |

**Table S3:** Performance of ablation experiments for each respiratory system diseases

| **Disease** | **TOP 1** | | | | | | | | | |
| --- | --- | --- | --- | --- | --- | --- | --- | --- | --- | --- |
|  | **Precision** | | | **Recall** | | | **F1-score** | | | |
|  | Base | Coarse | Standardization | Base | Coarse | Standardization | | Base | Coarse | Standardization |
| COPD | 0.577 | 0.639 | 0.669 | 0.936 | 0.932 | 0.914 | | 0.714 | 0.758 | 0.772 |
| bronchial asthma | 0.954 | 0.759 | 0.777 | 0.394 | 0.489 | 0.515 | | 0.558 | 0.594 | 0.62 |
| bronchiectasis | 0.883 | 0.701 | 0.731 | 0.322 | 0.550 | 0.598 | | 0.472 | 0.616 | 0.658 |
| airway stenosis | 0.808 | 0.717 | 0.746 | 0.545 | 0.429 | 0.610 | | 0.651 | 0.537 | 0.671 |
| pulmonary hypertension | 0.640 | 0.667 | 0.707 | 0.182 | 0.25 | 0.466 | | 0.283 | 0.364 | 0.562 |
| lung space-occupying lesions | 0.619 | 0.737 | 0.789 | 0.804 | 0.653 | 0.738 | | 0.700 | 0.692 | 0.763 |
| pulmonary infectious diseases | 0.609 | 0.673 | 0.646 | 0.498 | 0.541 | 0.531 | | 0.548 | 0.600 | 0.582 |
| pleural disease | 0.664 | 0.743 | 0.797 | 0.379 | 0.421 | 0.525 | | 0.483 | 0.538 | 0.633 |
| interstitial lung disease | 0.922 | 0.777 | 0.825 | 0.551 | 0.813 | 0.859 | | 0.690 | 0.795 | 0.842 |
|  | **TOP 3** | | | | | | | | | |
|  | **Precision** | | | **Recall** | | | | **F1-score** | | |
| COPD | 0.781 | 0.894 | 0.928 | 0.997 | 0.992 | 0.994 | | 0.876 | 0.941 | 0.960 |
| bronchial asthma | 0.988 | 0.947 | 0.960 | 0.625 | 0.746 | 0.826 | | 0.766 | 0.835 | 0.888 |
| bronchiectasis | 0.971 | 0.952 | 0.956 | 0.611 | 0.883 | 0.892 | | 0.750 | 0.917 | 0.923 |
| airway stenosis | 0.925 | 0.923 | 0.915 | 0.805 | 0.623 | 0.701 | | 0.861 | 0.744 | 0.794 |
| pulmonary hypertension | 0.932 | 0.870 | 0.879 | 0.466 | 0.455 | 0.580 | | 0.621 | 0.597 | 0.699 |
| lung space-occupying lesions | 0.810 | 0.950 | 0.950 | 0.910 | 0.910 | 0.941 | | 0.857 | 0.930 | 0.945 |
| pulmonary infectious diseases | 0.871 | 0.934 | 0.941 | 0.895 | 0.975 | 0.981 | | 0.883 | 0.954 | 0.961 |
| pleural disease | 0.943 | 0.956 | 0.978 | 0.946 | 0.743 | 0.847 | | 0.945 | 0.836 | 0.908 |
| interstitial lung disease | 0.983 | 0.928 | 0.971 | 0.684 | 0.942 | 0.957 | | 0.806 | 0.935 | 0.964 |

**Table S4:** Diagnostic performance for respiratory diseases of physicians, ***ChatGPT 4.0*** and the ***LungDiag***

| **LungDiag** | | | | | | |
| --- | --- | --- | --- | --- | --- | --- |
|  | TOP 1 | | | TOP 3 | | |
| Disease | precision | Recall | F1-score | precision | Recall | F1-score |
| COPD | 0.632 | 0.857 | 0.727 | 0.875 | 1.000 | 0.933 |
| bronchial asthma | 0.800 | 0.8 | 0.8 | 0.900 | 0.9 | 0.9 |
| bronchiectasis | 0.615 | 0.8 | 0.696 | 0.750 | 0.9 | 0.818 |
| airway stenosis | 1.000 | 0.625 | 0.769 | 1.000 | 0.625 | 0.769 |
| pulmonary hypertension | 0.778 | 0.7 | 0.737 | 0.909 | 1 | 0.952 |
| lung space-occupying lesions | 0.947 | 0.75 | 0.837 | 0.958 | 0.958 | 0.958 |
| pulmonary infectious diseases | 0.625 | 0.833 | 0.714 | 0.923 | 1 | 0.96 |
| pleural disease | 1.000 | 0.636 | 0.778 | 1 | 0.909 | 0.952 |
| interstitial lung disease | 0.750 | 0.818 | 0.783 | 1 | 0.818 | 0.9 |
| macro avg | 0.794 | 0.758 | 0.76 | 0.924 | 0.901 | 0.905 |

| **Physician 1** | | | | | | |
| --- | --- | --- | --- | --- | --- | --- |
| Disease | TOP 1 | | | TOP 3 | | |
| COPD | 0.343 | 0.857 | 0.49 | 0.619 | 0.929 | 0.743 |
| bronchial asthma | 0.583 | 0.7 | 0.636 | 0.8 | 0.8 | 0.8 |
| bronchiectasis | 0.625 | 0.5 | 0.556 | 0.9 | 0.9 | 0.9 |
| airway stenosis | 1 | 0.5 | 0.667 | 1 | 0.75 | 0.857 |
| pulmonary hypertension | 0.857 | 0.6 | 0.706 | 0.875 | 0.7 | 0.778 |
| lung space-occupying lesions | 0.706 | 0.5 | 0.585 | 0.913 | 0.875 | 0.894 |
| pulmonary infectious diseases | 0.571 | 0.333 | 0.421 | 0.818 | 0.75 | 0.783 |
| pleural disease | 0.2 | 0.091 | 0.125 | 0.9 | 0.818 | 0.857 |
| interstitial lung disease | 0.467 | 0.636 | 0.538 | 0.727 | 0.727 | 0.727 |
| macro avg | 0.595 | 0.524 | 0.525 | 0.839 | 0.805 | 0.815 |

| **Physician 2** | | | | | | |
| --- | --- | --- | --- | --- | --- | --- |
| Disease | TOP 1 | | | TOP 3 | | |
| COPD | 0.312 | 0.714 | 0.435 | 0.467 | 1 | 0.636 |
| bronchial asthma | 0.857 | 0.6 | 0.706 | 0.875 | 0.7 | 0.778 |
| bronchiectasis | 0.6 | 0.6 | 0.6 | 0.909 | 1 | 0.952 |
| airway stenosis | 1 | 0.75 | 0.857 | 1 | 1 | 1 |
| pulmonary hypertension | 0.375 | 0.3 | 0.333 | 0.667 | 0.6 | 0.632 |
| lung space-occupying lesions | 0.714 | 0.625 | 0.667 | 0.9 | 0.75 | 0.818 |
| pulmonary infectious diseases | 0.333 | 0.5 | 0.4 | 0.533 | 0.667 | 0.593 |
| pleural disease | 1 | 0.182 | 0.308 | 1 | 0.273 | 0.429 |
| interstitial lung disease | 0.167 | 0.091 | 0.118 | 0.667 | 0.364 | 0.471 |
| macro avg | 0.595 | 0.485 | 0.491 | 0.78 | 0.706 | 0.701 |

| **Physician 3** | | | | | | |
| --- | --- | --- | --- | --- | --- | --- |
| Disease | TOP 1 | | | TOP 3 | | |
| COPD | 0.429 | 0.429 | 0.429 | 0.765 | 0.929 | 0.839 |
| bronchial asthma | 0.75 | 0.6 | 0.667 | 0.875 | 0.7 | 0.778 |
| bronchiectasis | 0.25 | 0.2 | 0.222 | 0.667 | 0.6 | 0.632 |
| airway stenosis | 1 | 0.125 | 0.222 | 1 | 0.75 | 0.857 |
| pulmonary hypertension | 0.562 | 0.9 | 0.692 | 0.692 | 0.9 | 0.783 |
| lung space-occupying lesions | 0.636 | 0.583 | 0.609 | 0.87 | 0.833 | 0.851 |
| pulmonary infectious diseases | 0.316 | 0.5 | 0.387 | 0.846 | 0.917 | 0.88 |
| pleural disease | 0.75 | 0.273 | 0.4 | 1 | 0.818 | 0.9 |
| interstitial lung disease | 0.444 | 0.727 | 0.552 | 0.833 | 0.909 | 0.87 |
| macro avg | 0.571 | 0.482 | 0.464 | 0.839 | 0.817 | 0.821 |

| **Physician 4** | | | | | | |
| --- | --- | --- | --- | --- | --- | --- |
| Disease | TOP 1 | | | TOP 3 | | |
| COPD | 0.263 | 0.714 | 0.385 | 0.424 | 1 | 0.596 |
| bronchial asthma | 1 | 0.6 | 0.75 | 1 | 0.7 | 0.824 |
| bronchiectasis | 0.667 | 0.6 | 0.632 | 0.833 | 1 | 0.909 |
| airway stenosis | 1 | 0.75 | 0.857 | 1 | 1 | 1 |
| pulmonary hypertension | 0.625 | 0.5 | 0.556 | 0.875 | 0.7 | 0.778 |
| lung space-occupying lesions | 0.652 | 0.625 | 0.638 | 0.85 | 0.708 | 0.773 |
| pulmonary infectious diseases | 0.412 | 0.583 | 0.483 | 0.688 | 0.917 | 0.786 |
| pleural disease | 1 | 0.091 | 0.167 | 1 | 0.182 | 0.308 |
| interstitial lung disease | 0.5 | 0.091 | 0.154 | 1 | 0.364 | 0.533 |
| macro avg | 0.68 | 0.506 | 0.513 | 0.852 | 0.73 | 0.723 |

| **Physician 5** | | | | | | |
| --- | --- | --- | --- | --- | --- | --- |
| Disease | TOP 1 | | | TOP 3 | | |
| COPD | 0.296 | 0.571 | 0.39 | 0.667 | 0.857 | 0.75 |
| bronchial asthma | 0.667 | 0.2 | 0.308 | 0.875 | 0.7 | 0.778 |
| bronchiectasis | 0.333 | 0.1 | 0.154 | 0.875 | 0.7 | 0.778 |
| airway stenosis | 0.667 | 0.25 | 0.364 | 1 | 0.875 | 0.933 |
| pulmonary hypertension | 0.889 | 0.8 | 0.842 | 1 | 0.9 | 0.947 |
| lung space-occupying lesions | 0.708 | 0.708 | 0.708 | 0.917 | 0.917 | 0.917 |
| pulmonary infectious diseases | 0.296 | 0.667 | 0.41 | 0.667 | 1 | 0.8 |
| pleural disease | 1 | 0.364 | 0.533 | 1 | 0.727 | 0.842 |
| interstitial lung disease | 0.4 | 0.364 | 0.381 | 0.8 | 0.727 | 0.762 |
| macro avg | 0.584 | 0.447 | 0.454 | 0.867 | 0.823 | 0.834 |

| **Physician 6** | | | | | | |
| --- | --- | --- | --- | --- | --- | --- |
| Disease | TOP 1 | | | TOP 3 | | |
| COPD | 0.276 | 0.571 | 0.372 | 0.5 | 1 | 0.667 |
| bronchial asthma | 0.857 | 0.6 | 0.706 | 0.875 | 0.7 | 0.778 |
| bronchiectasis | 0.6 | 0.6 | 0.6 | 1 | 1 | 1 |
| airway stenosis | 0.857 | 0.75 | 0.8 | 1 | 1 | 1 |
| pulmonary hypertension | 0.4 | 0.4 | 0.4 | 0.636 | 0.7 | 0.667 |
| lung space-occupying lesions | 0.7 | 0.583 | 0.636 | 0.909 | 0.833 | 0.87 |
| pulmonary infectious diseases | 0.222 | 0.333 | 0.267 | 0.538 | 0.583 | 0.56 |
| pleural disease | 1 | 0.091 | 0.167 | 1 | 0.273 | 0.429 |
| interstitial lung disease | 0.125 | 0.091 | 0.105 | 0.571 | 0.364 | 0.444 |
| macro avg | 0.56 | 0.447 | 0.45 | 0.781 | 0.717 | 0.713 |

| **Physician 7** | | | | | | |
| --- | --- | --- | --- | --- | --- | --- |
| Disease | TOP 1 | | | TOP 3 | | |
| COPD | 0.385 | 0.714 | 0.5 | 0.55 | 0.786 | 0.647 |
| bronchial asthma | 0.545 | 0.6 | 0.571 | 0.583 | 0.7 | 0.636 |
| bronchiectasis | 0.6 | 0.3 | 0.4 | 0.857 | 0.6 | 0.706 |
| airway stenosis | 0.8 | 0.5 | 0.615 | 0.875 | 0.875 | 0.875 |
| pulmonary hypertension | 0.412 | 0.7 | 0.519 | 0.7 | 0.7 | 0.7 |
| lung space-occupying lesions | 0.739 | 0.708 | 0.723 | 0.92 | 0.958 | 0.939 |
| pulmonary infectious diseases | 0.7 | 0.583 | 0.636 | 0.857 | 1 | 0.923 |
| pleural disease | 1 | 0.273 | 0.429 | 1 | 0.545 | 0.706 |
| interstitial lung disease | 0.5 | 0.455 | 0.476 | 0.75 | 0.545 | 0.632 |
| macro avg | 0.631 | 0.537 | 0.541 | 0.788 | 0.746 | 0.752 |

| **Physician 8** | | | | | | |
| --- | --- | --- | --- | --- | --- | --- |
| Disease | TOP 1 | | | TOP 3 | | |
| COPD | 0.385 | 0.714 | 0.5 | 0.476 | 0.714 | 0.571 |
| bronchial asthma | 0.5 | 0.6 | 0.545 | 0.5 | 0.6 | 0.545 |
| bronchiectasis | 0.571 | 0.4 | 0.471 | 0.833 | 0.5 | 0.625 |
| airway stenosis | 0.667 | 0.5 | 0.571 | 0.875 | 0.875 | 0.875 |
| pulmonary hypertension | 0.467 | 0.7 | 0.56 | 0.667 | 0.8 | 0.727 |
| lung space-occupying lesions | 0.739 | 0.708 | 0.723 | 0.958 | 0.958 | 0.958 |
| pulmonary infectious diseases | 0.556 | 0.417 | 0.476 | 0.846 | 0.917 | 0.88 |
| pleural disease | 1 | 0.182 | 0.308 | 1 | 0.636 | 0.778 |
| interstitial lung disease | 0.5 | 0.455 | 0.476 | 0.714 | 0.455 | 0.556 |
| macro avg | 0.598 | 0.52 | 0.515 | 0.763 | 0.717 | 0.724 |

| **Physician 9** | | | | | | |
| --- | --- | --- | --- | --- | --- | --- |
| Disease | TOP 1 | | | TOP 3 | | |
| COPD | 0.278 | 0.714 | 0.4 | 0.433 | 0.929 | 0.591 |
| bronchial asthma | 0.8 | 0.4 | 0.533 | 1 | 0.4 | 0.571 |
| bronchiectasis | 0.5 | 0.1 | 0.167 | 0.8 | 0.4 | 0.533 |
| airway stenosis | 1 | 0.25 | 0.4 | 1 | 0.625 | 0.769 |
| pulmonary hypertension | 0.5 | 0.8 | 0.615 | 0.714 | 1 | 0.833 |
| lung space-occupying lesions | 0.692 | 0.75 | 0.72 | 0.778 | 0.875 | 0.824 |
| pulmonary infectious diseases | 0.375 | 0.5 | 0.429 | 0.667 | 0.833 | 0.741 |
| pleural disease | 1 | 0.091 | 0.167 | 1 | 0.273 | 0.429 |
| interstitial lung disease | 0.667 | 0.364 | 0.471 | 0.714 | 0.455 | 0.556 |
| macro avg | 0.646 | 0.441 | 0.433 | 0.79 | 0.643 | 0.65 |

| **ChatGPT 4.0** | | | | | | |
| --- | --- | --- | --- | --- | --- | --- |
| Disease | TOP 1 | | | TOP 3 | | |
| COPD | 0.289 | 0.786 | 0.423 | 0.565 | 0.929 | 0.703 |
| bronchial asthma | 1 | 0.5 | 0.667 | 1 | 0.9 | 0.947 |
| bronchiectasis | 0.333 | 0.4 | 0.364 | 0.6 | 0.6 | 0.6 |
| airway stenosis | 1 | 0.125 | 0.222 | 1 | 0.75 | 0.857 |
| pulmonary hypertension | 1 | 0.6 | 0.75 | 1 | 0.9 | 0.947 |
| lung space-occupying lesions | 0.684 | 0.542 | 0.605 | 0.9 | 0.75 | 0.818 |
| pulmonary infectious diseases | 0.227 | 0.417 | 0.294 | 0.632 | 1 | 0.774 |
| pleural disease | 1 | 0.091 | 0.167 | 1 | 0.364 | 0.533 |
| interstitial lung disease | 0.667 | 0.364 | 0.471 | 0.8 | 0.727 | 0.762 |
| macro avg | 0.689 | 0.425 | 0.44 | 0.833 | 0.769 | 0.771 |

**Table S5:** Comparison of diagnostic performance of physicians, ***ChatGPT 4.0*** and the ***LungDiag***

| **Disease** | **TOP 1** | | | **TOP 3** | | |
| --- | --- | --- | --- | --- | --- | --- |
|  | Physician | **ChatGPT** | ***LungDiag*** | Physician | **ChatGPT** | ***LungDiag*** |
| COPD | 0.434 | 0.423 | 0.686 | 0.698 | 0.703 | 0.903 |
| Bronchial asthma | 0.602 | 0.667 | 0.762 | 0.716 | 0.947 | 0.909 |
| Bronchiectasis | 0.422 | 0.364 | 0.762 | 0.751 | 0.600 | 0.857 |
| Airway stenosis | 0.595 | 0.222 | 0.769 | 0.891 | 0.857 | 0.857 |
| Pulmonary hypertension | 0.580 | 0.750 | 0.800 | 0.761 | 0.947 | 1.000 |
| Lung space-occupying lesions | 0.668 | 0.605 | 0.810 | 0.880 | 0.818 | 0.913 |
| Pulmonary infectious diseases | 0.434 | 0.294 | 0.667 | 0.782 | 0.774 | 1.000 |
| Pleural disease | 0.289 | 0.167 | 0.667 | 0.697 | 0.533 | 0.952 |
| Interstitial lung disease | 0.363 | 0.471 | 0.783 | 0.654 | 0.762 | 0.952 |

We used the average F1-score to evaluate the diagnosis performance across different groups.

**Table S6:** Hyperparameters of the ***LungDiag*** model

| ***LungDiag*** | **Hyperparameters** | **Value** |
| --- | --- | --- |
| BiLSTM-CRF | Sequence length | 300 |
|  | Epochs | 30 |
|  | Early stopping patience | 10 |
|  | Batch size | 64 |
| LightGBM | Boosting type | gbdt |
|  | Colsample bytree | 1.0 |
|  | Learning rate | 0.1 |
|  | Max depth | -1 |
|  | Min child samples | 20 |
|  | Min child weight | 0.001 |
|  | Min split gain | 0.0 |
|  | N_estimators | 100 |
|  | Num class | 10 |

**Table S7:** Definition and typical values of the attribute

| **Attribute** | **Definition** | **Typical values** |
| --- | --- | --- |
| Assertion  (sct: 260245000) | A physician’s belief status with regards to a particular patient’s medical problem. The assertion is reflected in two aspects: whether the entity occurs to the patient, and how the entity occurs to the patient. | Present  (sct:52101004);  Absent  (sct:2667000);  H/O: Disorder  (sct: 312850006);  Possible  (sct:371930009);  Family history of disorder  (sct: 281666001) |
| Severity of phenotypes  (sct: 272141005) | The intensity or degree of a manifestation. | Mild  (sct: 255604002);  Moderate  (sct: 6736007);  Severe  (sct: 24484000);  Life threatening severity  (sct: 442452003) |
| Pain by sensation quality  (sct: 410720000) | A subjective category or type of Pain by sensation quality. | Dull  (sct: 263744001);  Tender  (sct: 300820003);  Soreness  (sct: 71393004);  Sharp  (sct: 410707003);  Colicky pain  (sct: 73063007);  Stabbing pain  (sct: 55145008);  Electric shock type pain  (sct: 723316007);  Splitting pain  (sct: 279097006);  Cutting pain  (sct: 162503007); |
| Temporal pattern  (sct: 272103003) | The speed at which disease manifestations appear and develop. | Acute  (sct: 272118002);  Subacute  (sct: 19939008);  Chronic  (sct: 90734009);  Periodic  (sct: 81591007);  Episodic  (sct: 278499009);  Transient  (sct: 14803004);  Migratory  (sct: 255309000);  Insidious onset  (sct: 367326009);  Recurrent  (sct: 255227004);  Prolonged  (sct:255224006) |
| Symptom aggravating factors  (sct: 162473008) | Something that makes a condition worse. | Movement aggravates symptom  (sct: 162477009)  Cold aggravates symptom  (sct: 162479007) |
| Symptom relieving factors  (sct: 162483007) | Something that relieve the condition. | Rest relieves symptom  (sct: 162488003)  Food relieves symptom  (sct: 162485000) |
| Spatial pattern  (sct: 255464007) | The pattern by which a phenotype affects one or more regions of the body. | Generalized  (sct: 60132005);  Localized  (sct: 255471002);  Diffuse  (sct: 19648000) |
| Laterality  (sct: 272741003) | The localization with respect to the side of the body of the specified phenotypic abnormality. | Left  (sct: 7771000);  Right  (sct: 24028007);  Unilateral  (sct: 66459002);  Bilateral  (sct: 51440002) |
| Quadrant pattern  (sct: 272137006) | The pattern by which a phenotype affects the four quadrants of abdomen or breast. | Left upper quadrant  (sct: 255481003);  Left lower quadrant  (sct: 255480002);  Right upper quadrant  (sct: 255497008);  Right lower quadrant  (sct: 255495000) |
| Specimen  (sct: 123038009) | A portion or quantity of material for use in testing, examination, or study. | Blood specimen  (sct: 119297000);  Urine specimen  (sct: 122575003);  Faeces specimen  (sct:119339001);  Sputum specimen  (sct: 119334006);  Saliva specimen  (sct: 119342007) |

| **A** | **B** |
| --- | --- |
| **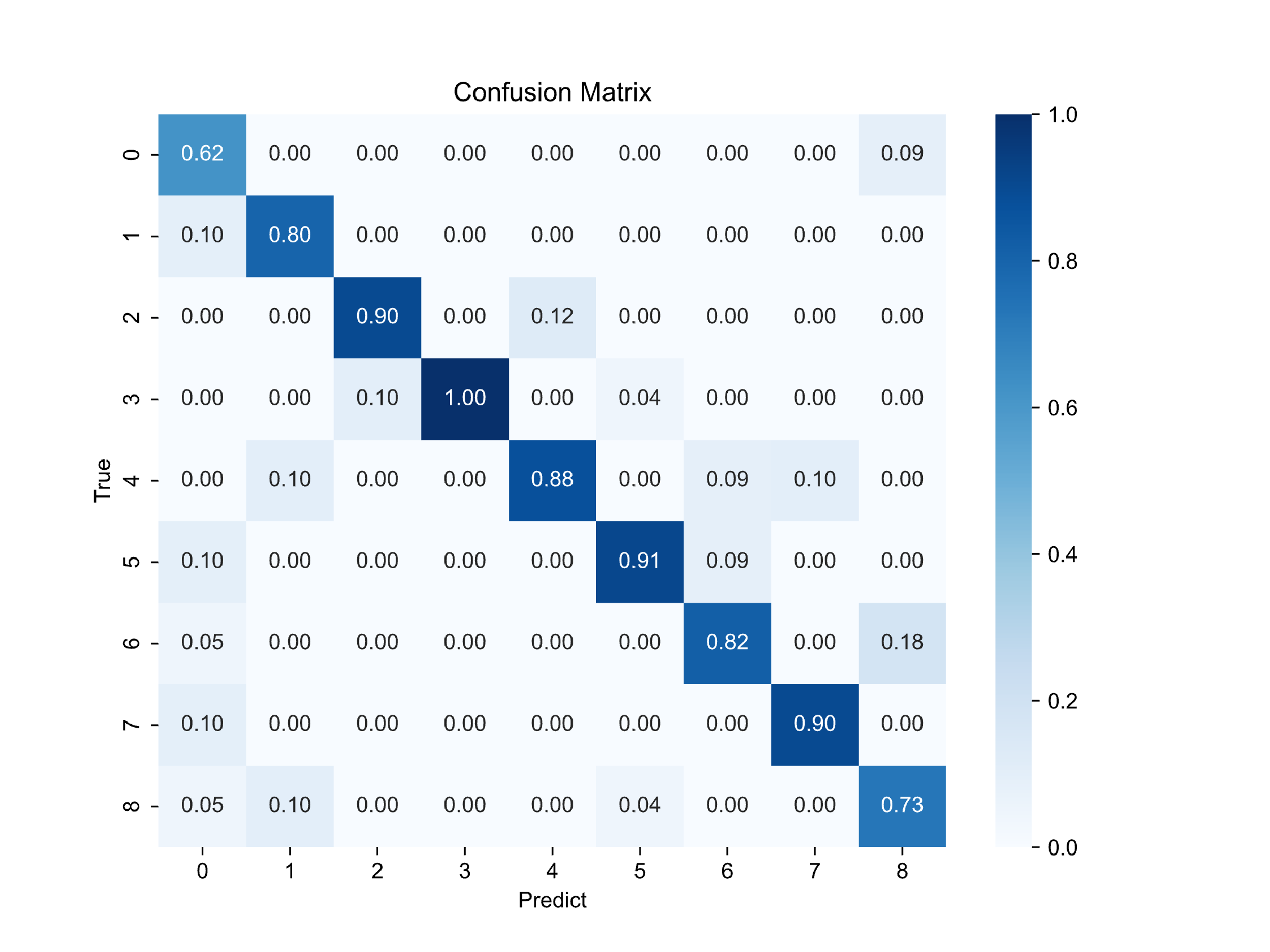** | **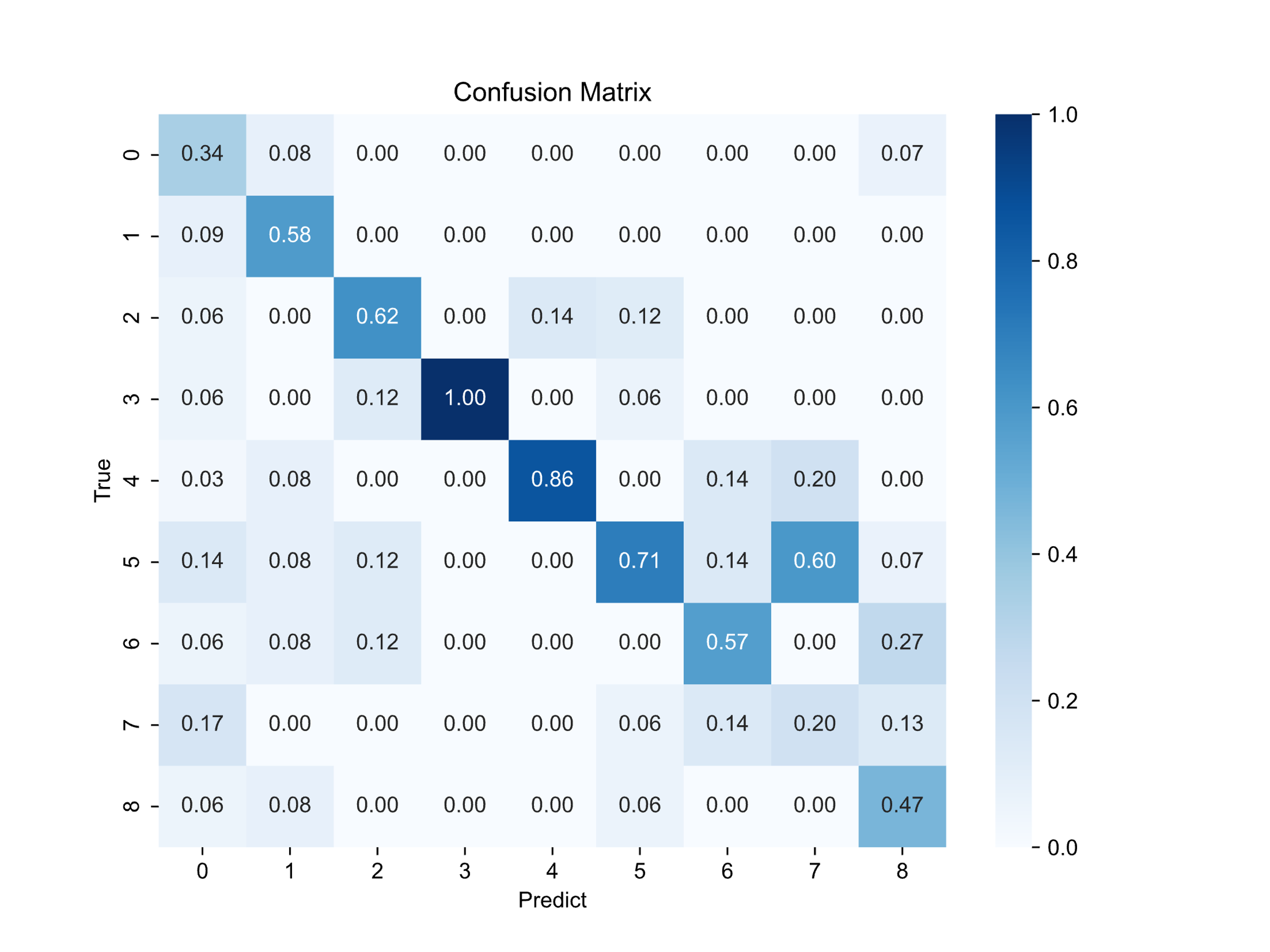** |
| **C** | **D** |
| **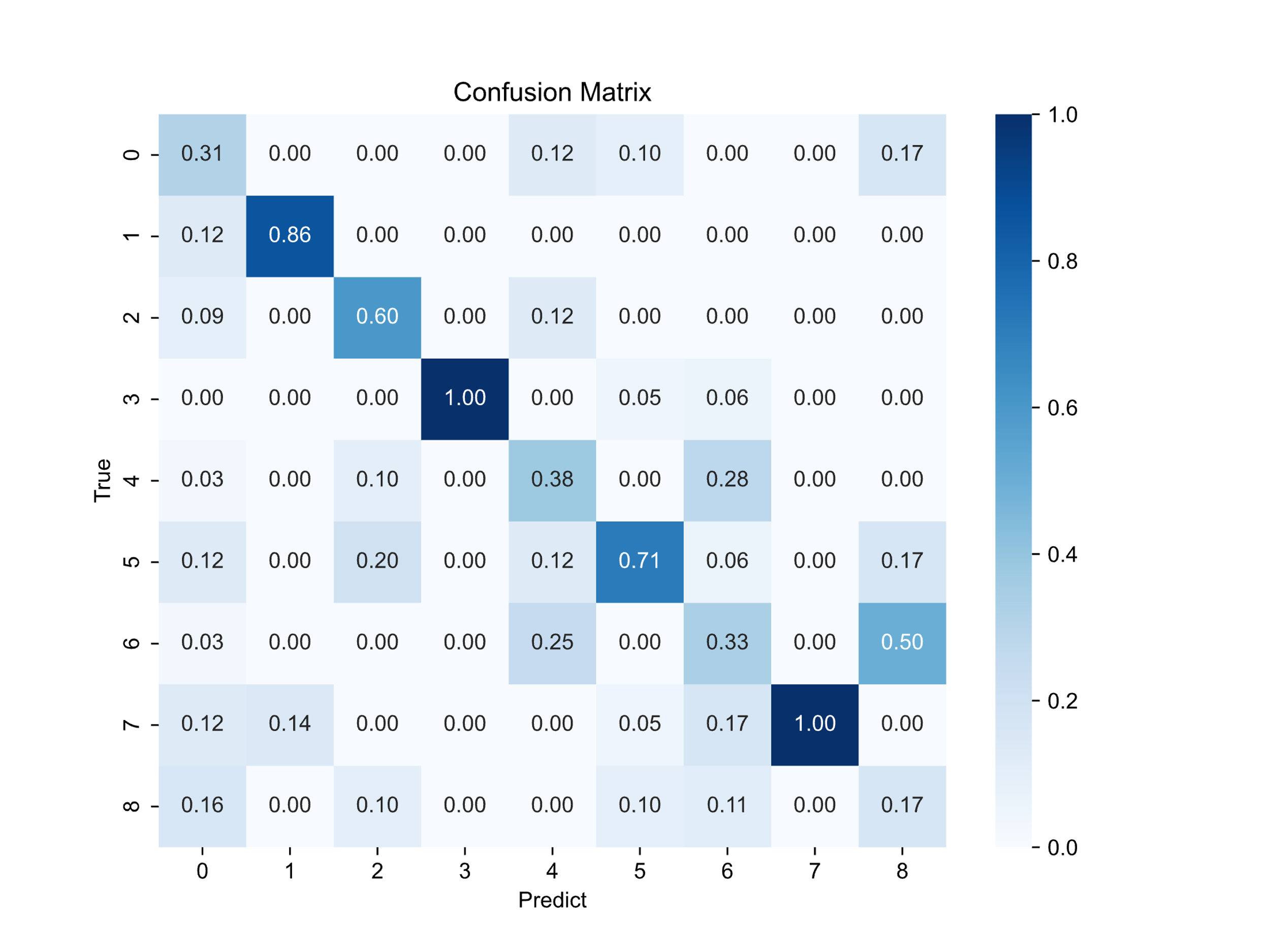** | **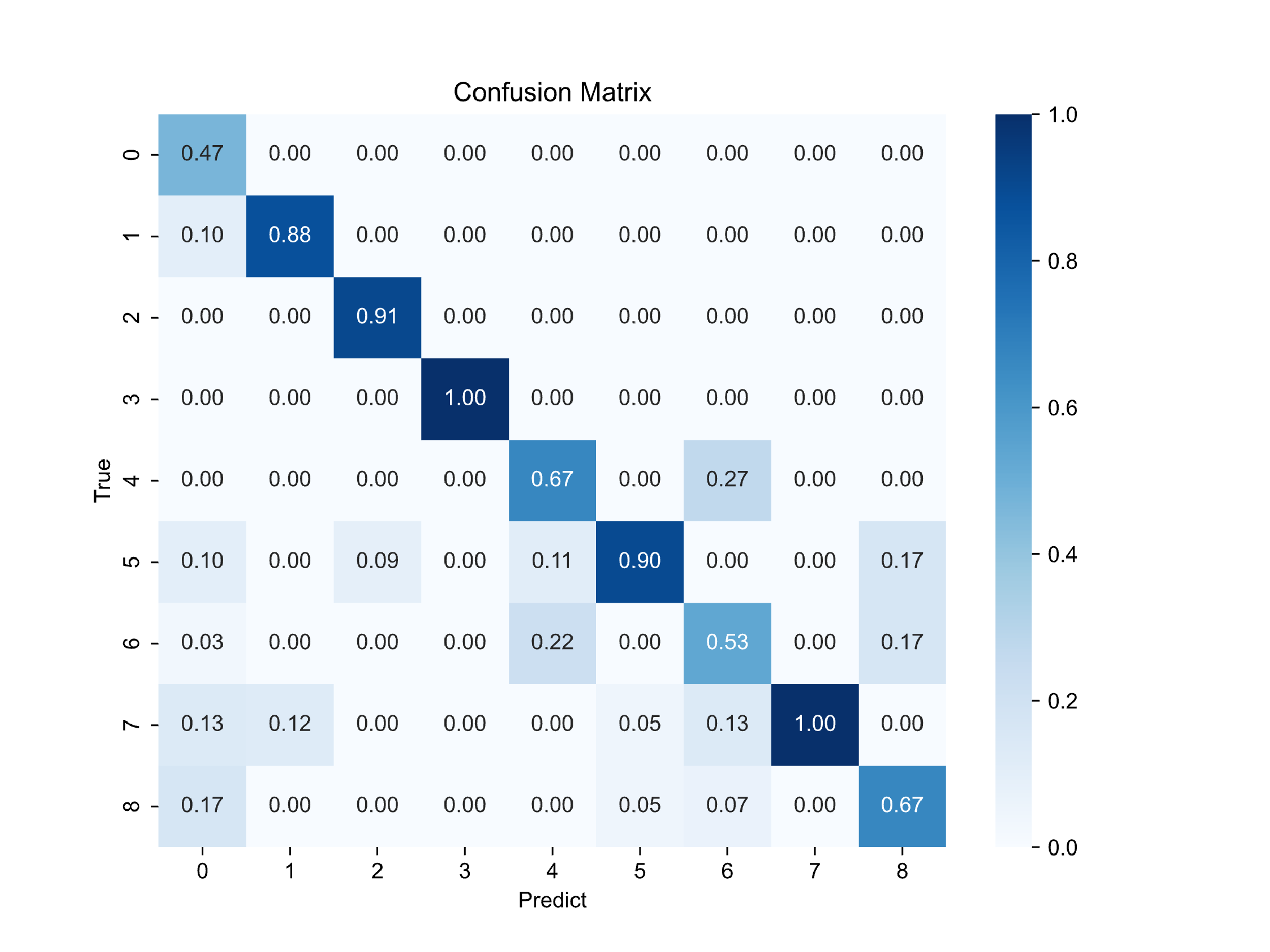** |
| **E** | **F** |
| **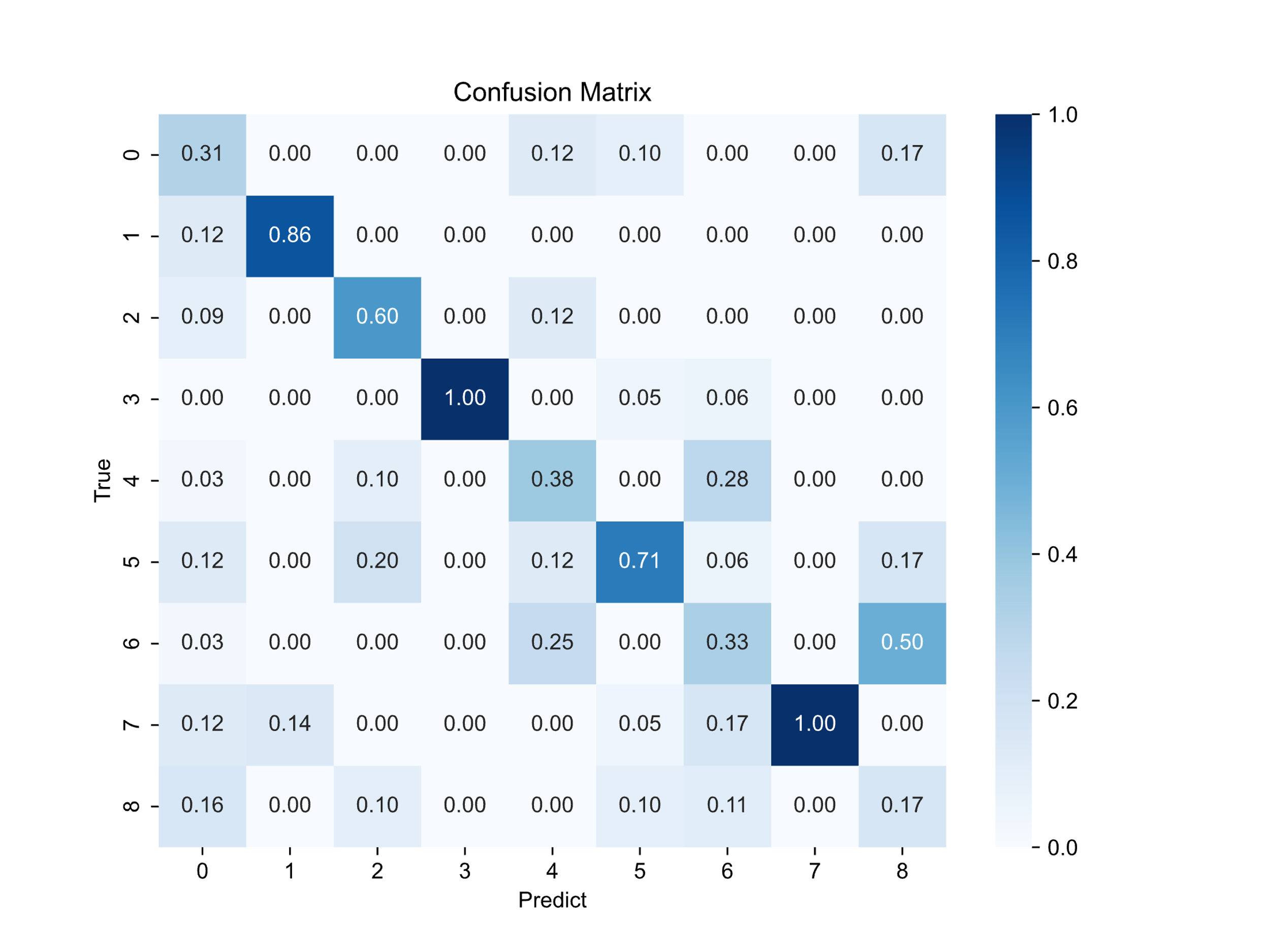** | **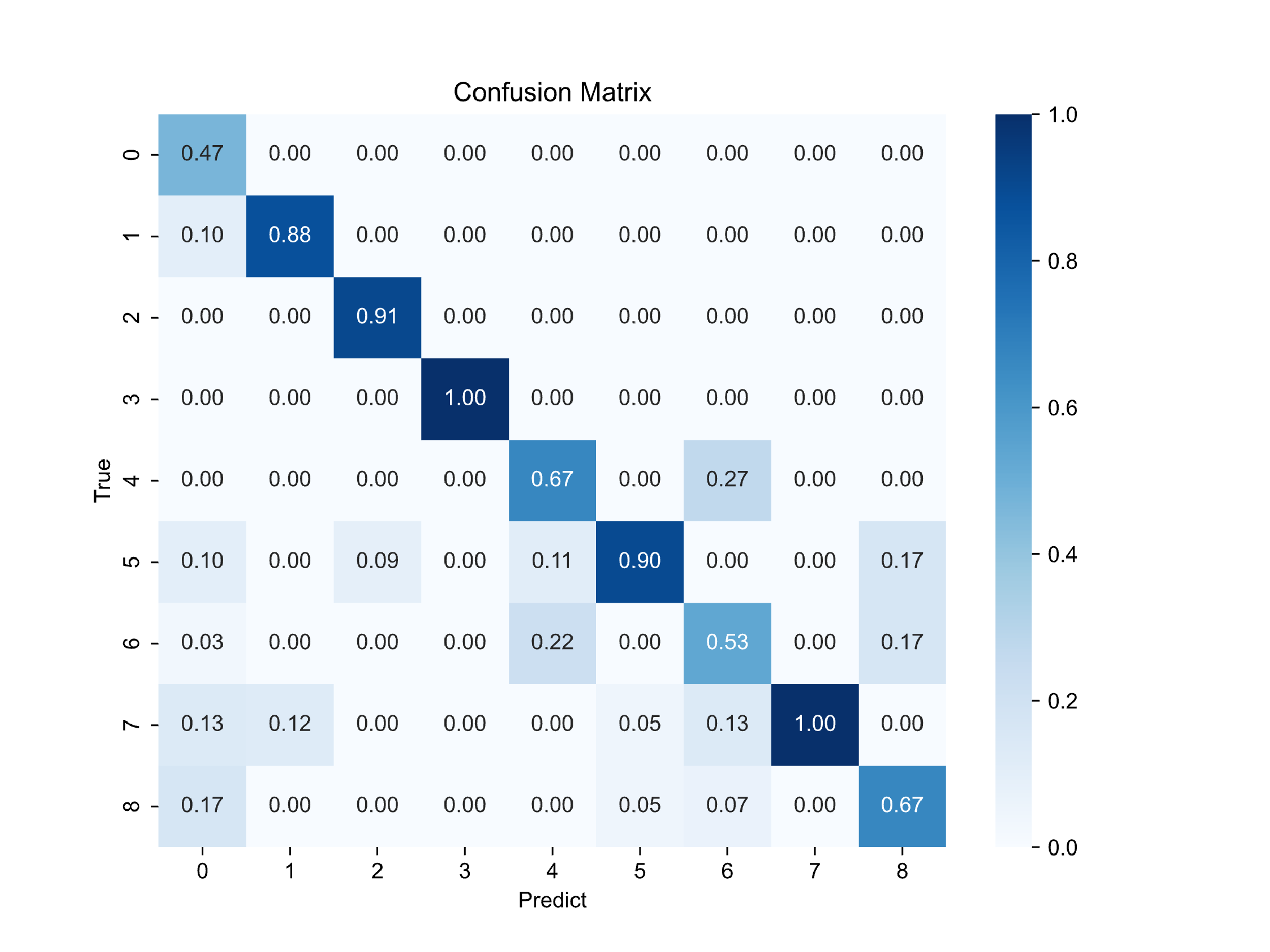** |

| **G** | **H** |
| --- | --- |
| **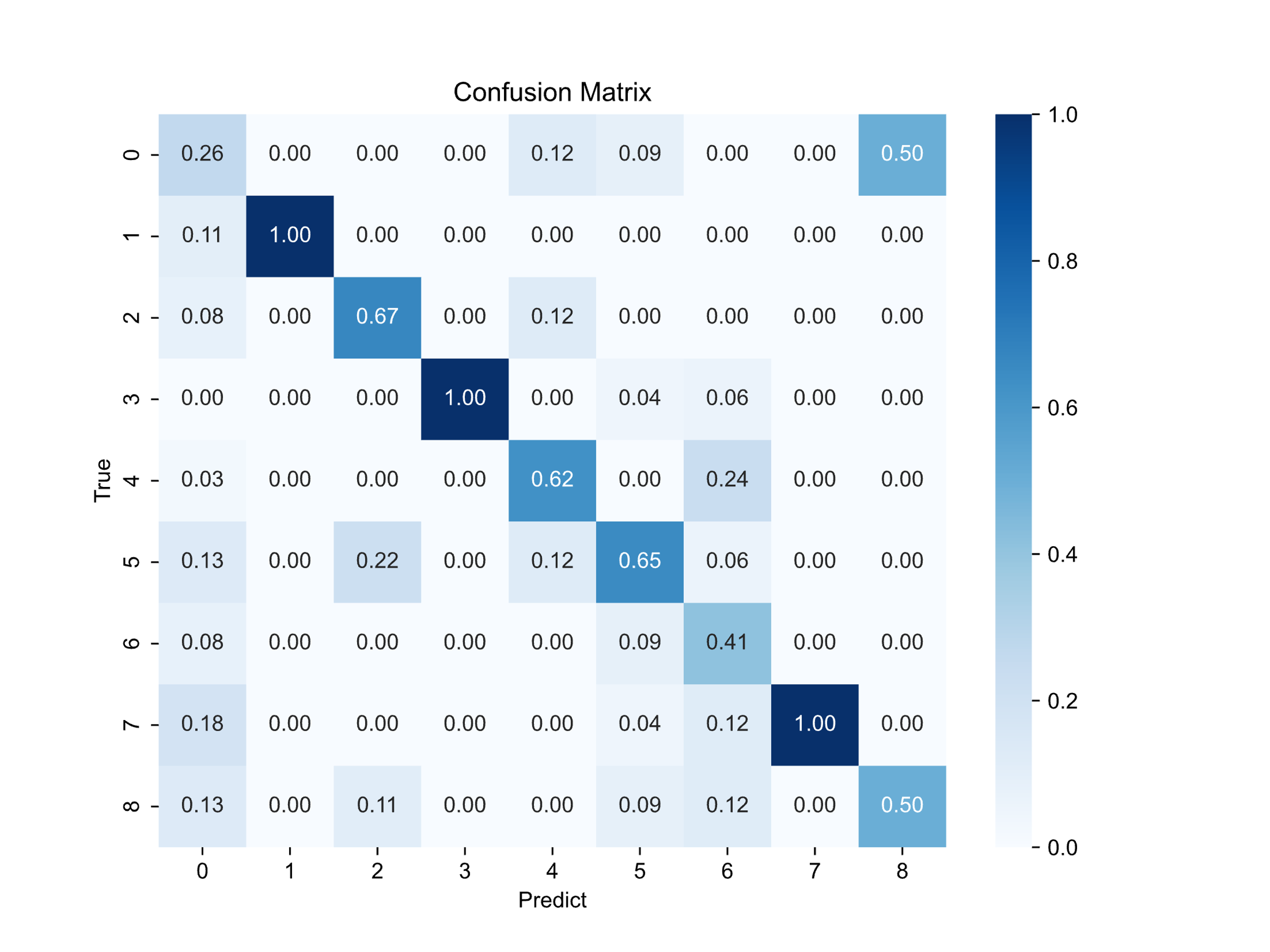** | **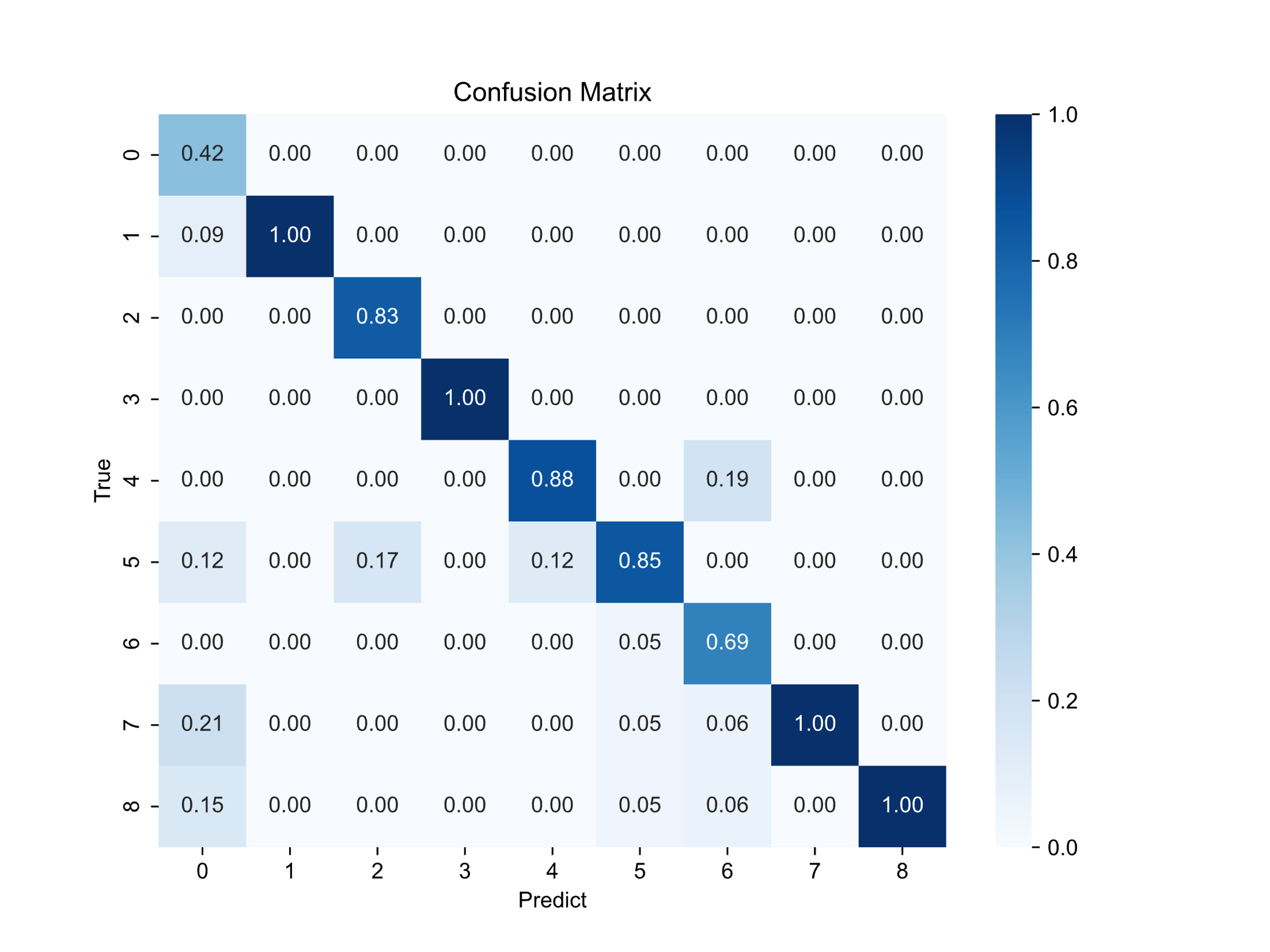** |
| **I** | **J** |
| **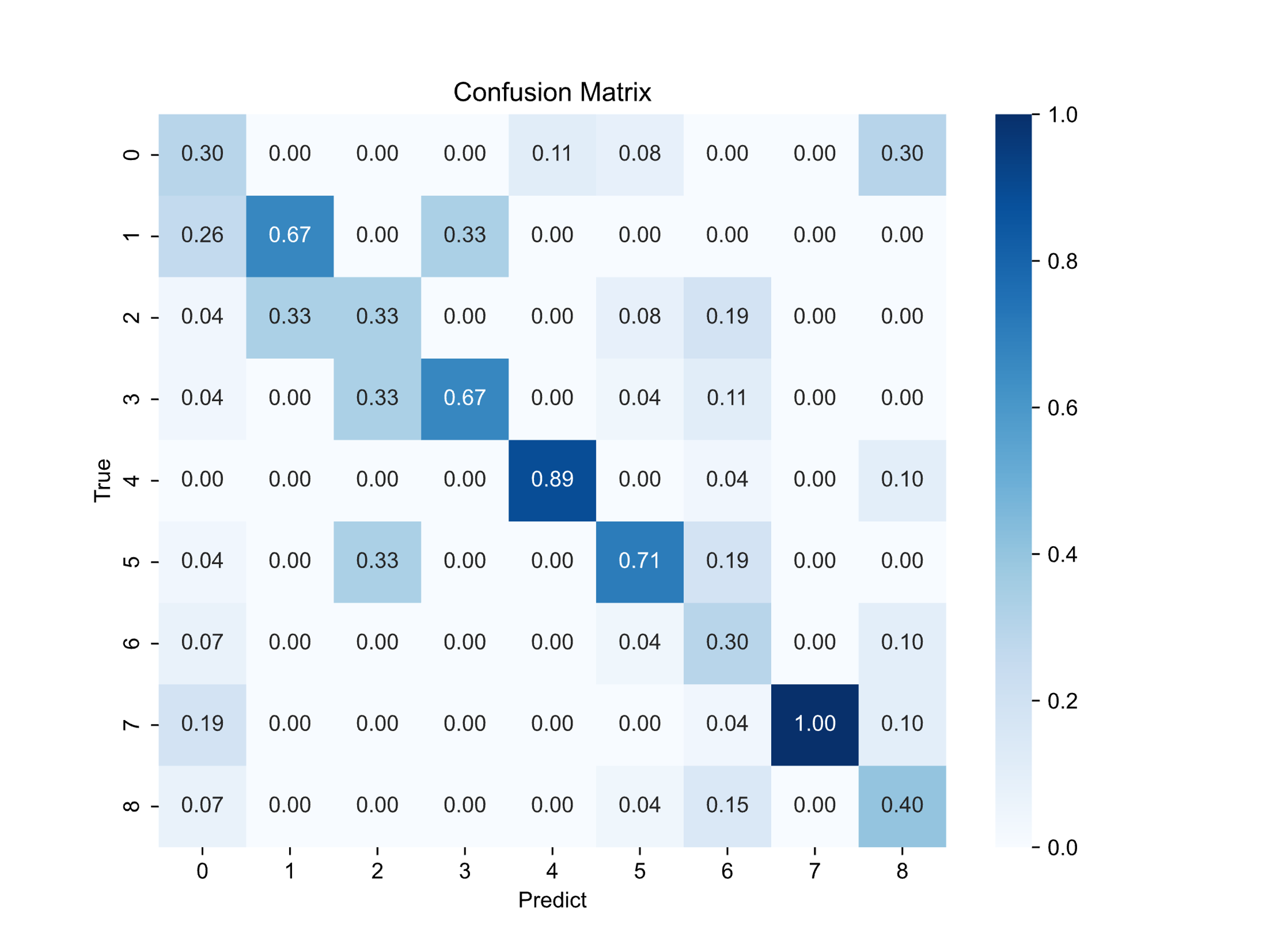** | **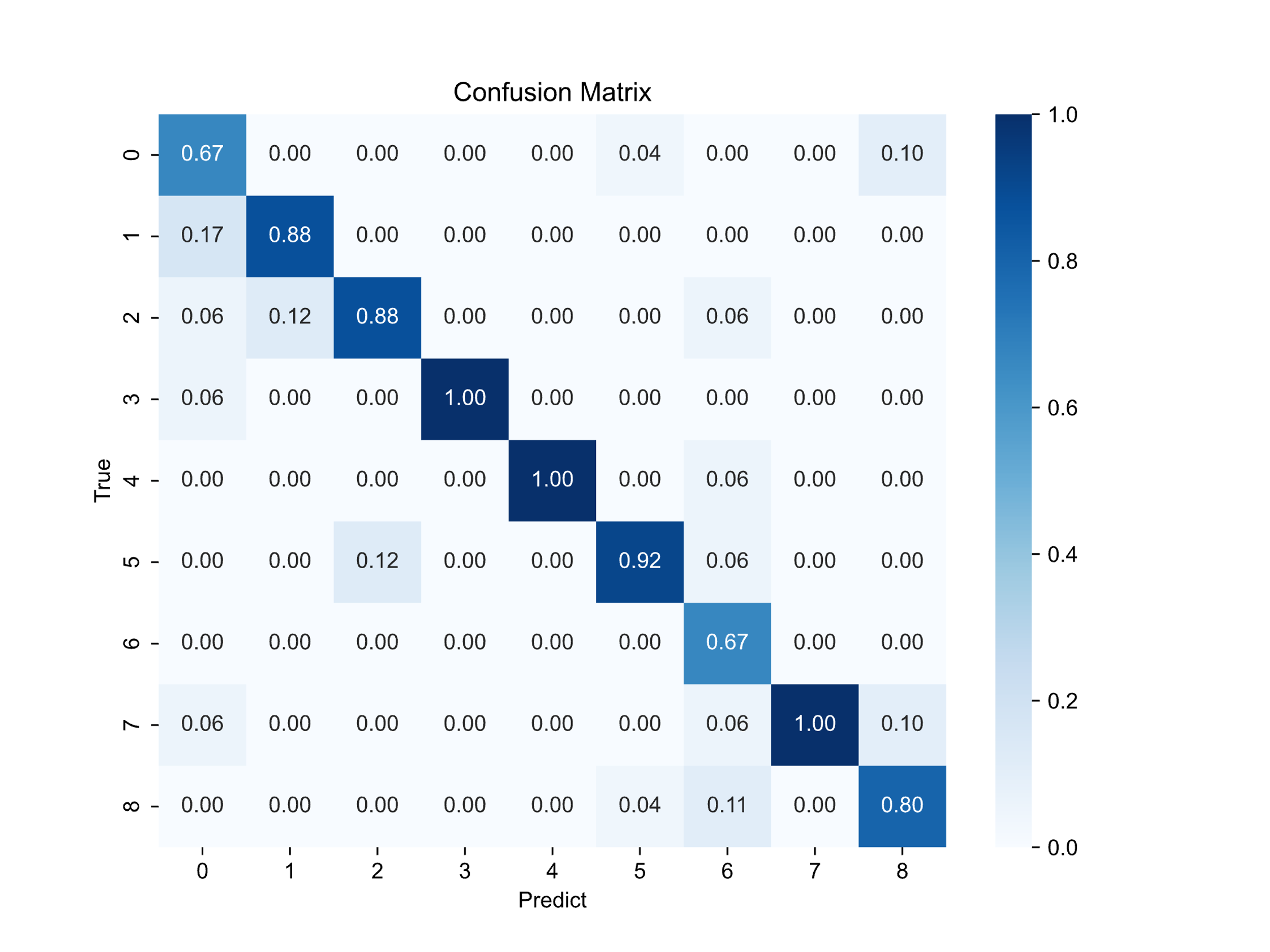** |
| **K** | **L** |
| **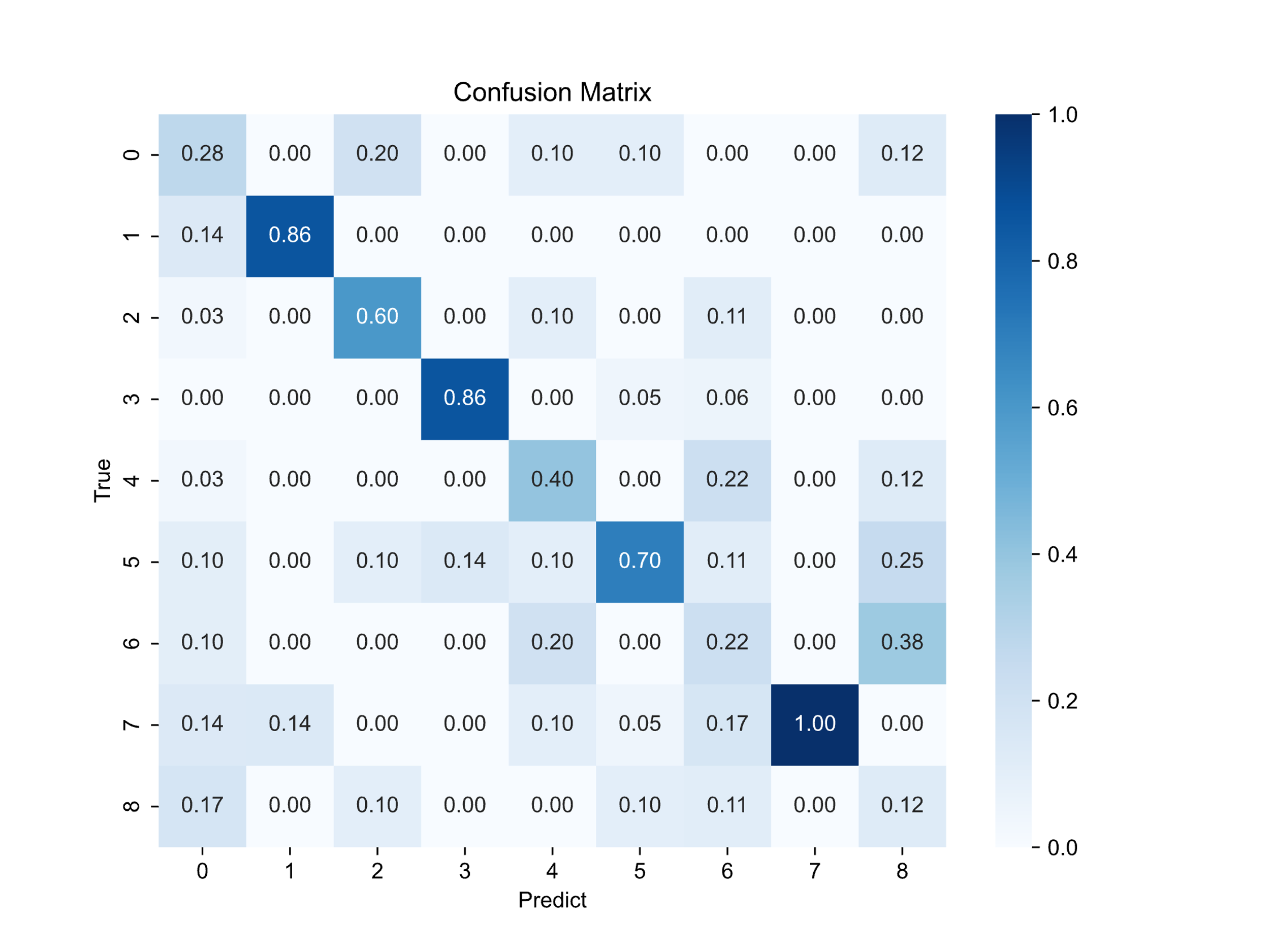** | **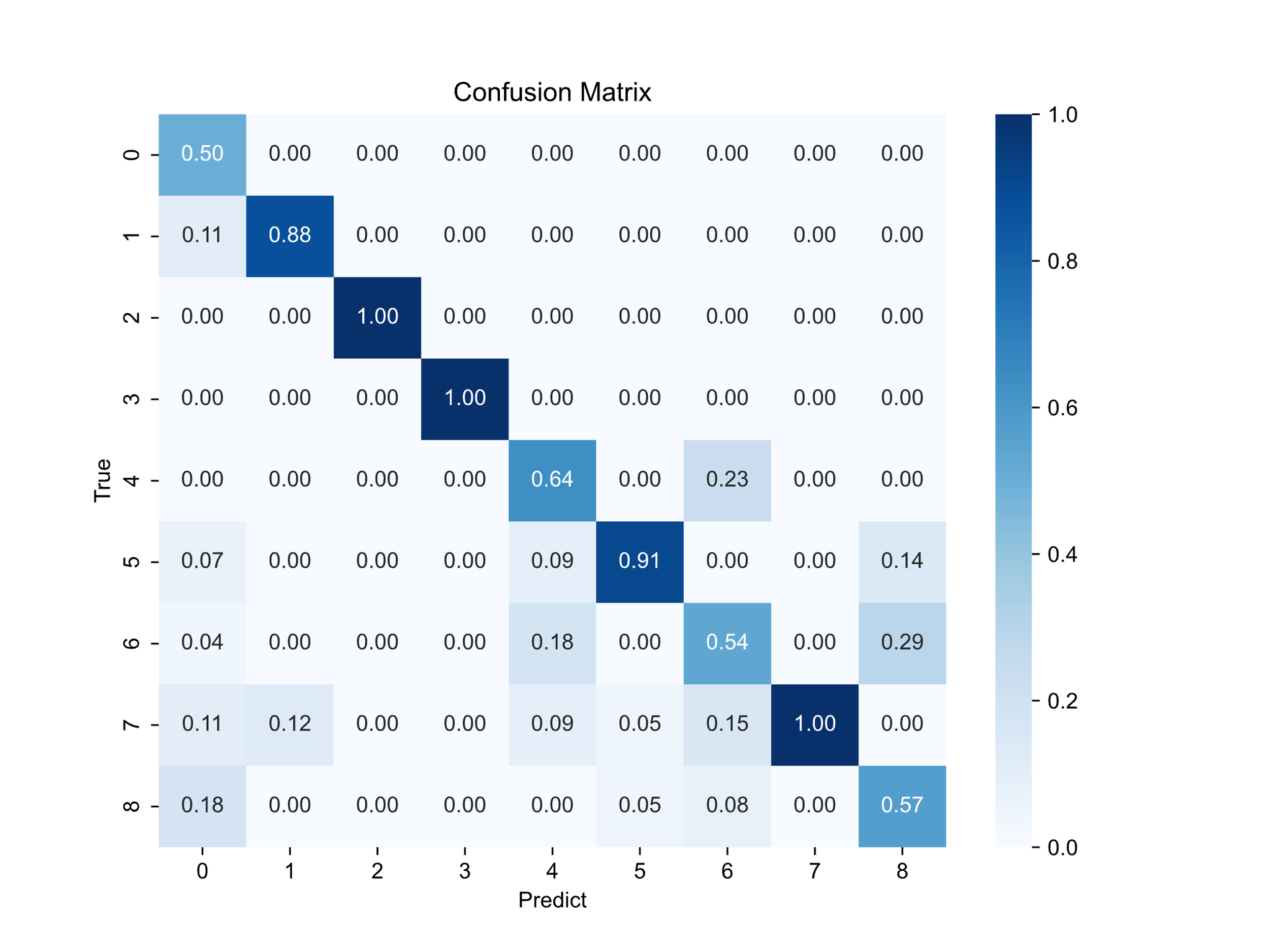** |

| **M** | **N** |
| --- | --- |
| **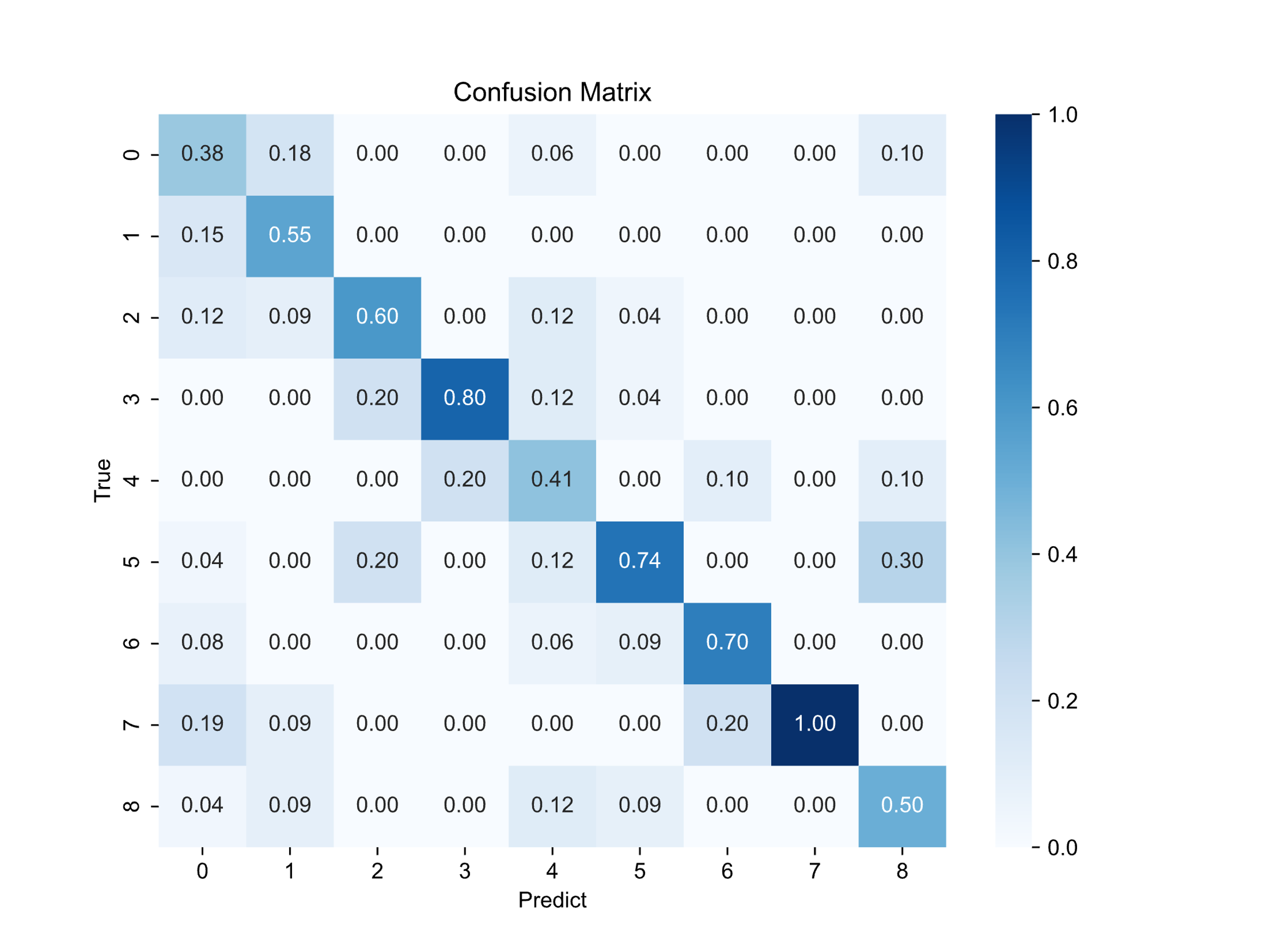** | **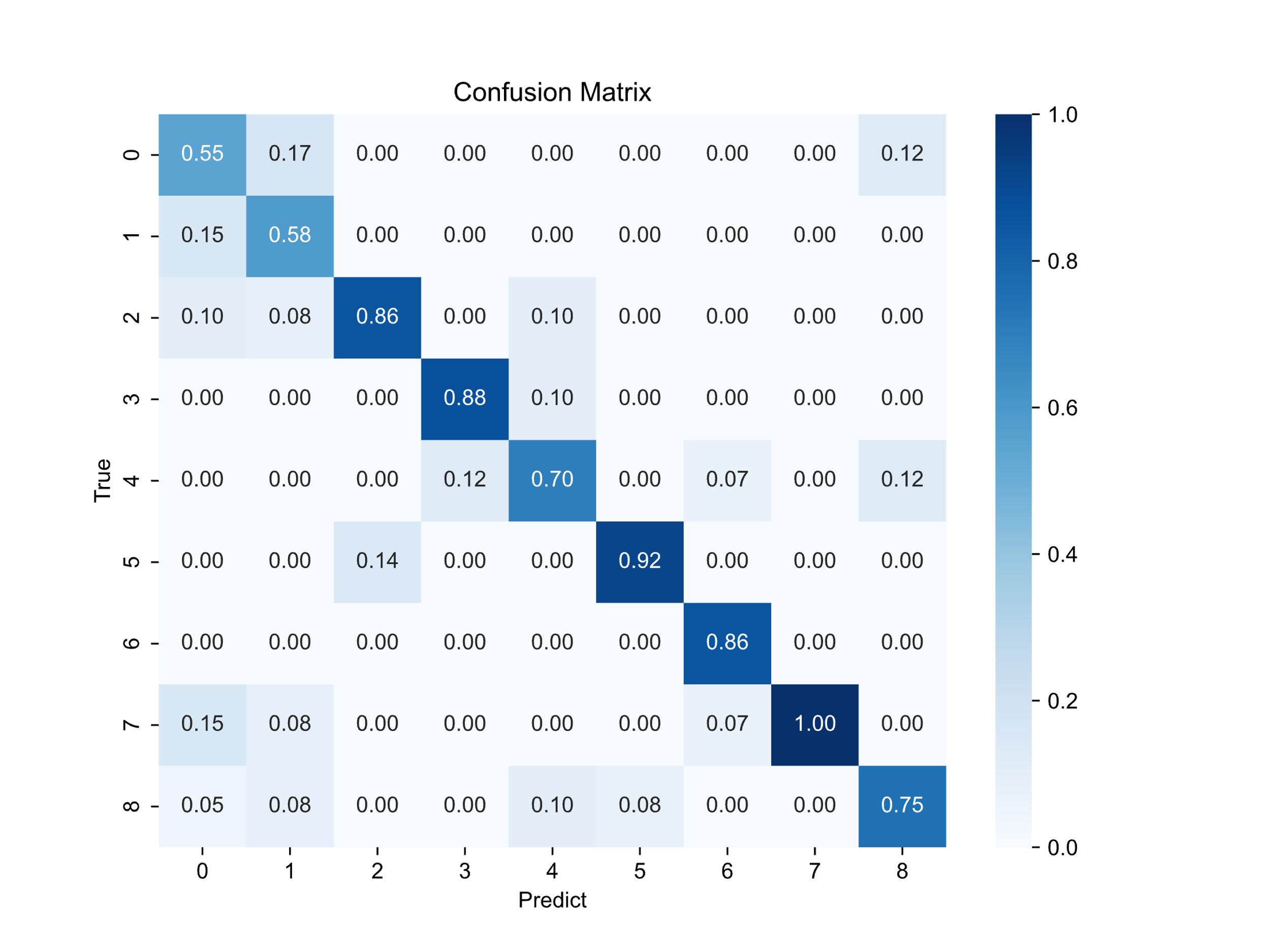** |
| **O** | **P** |
| **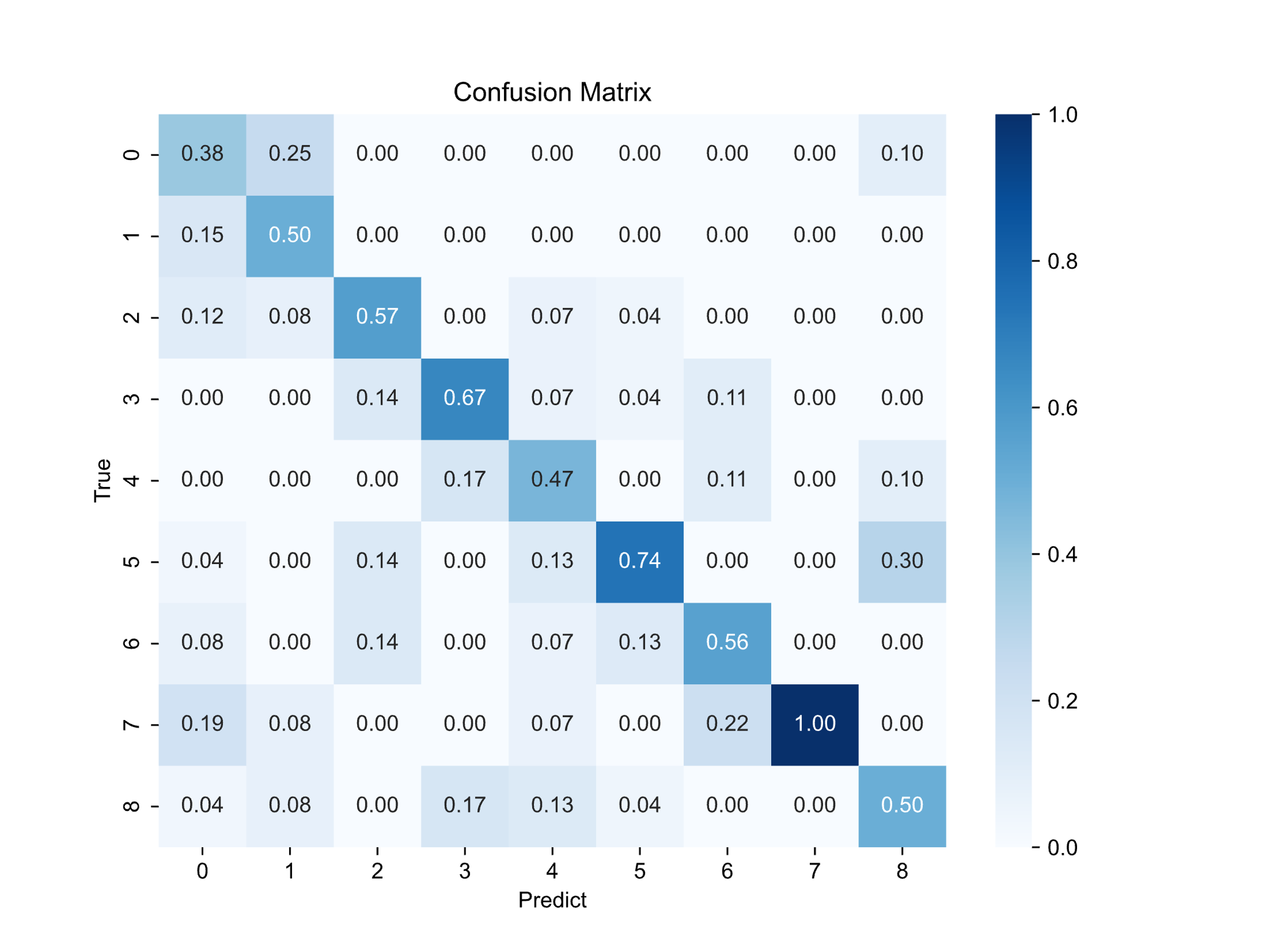** | **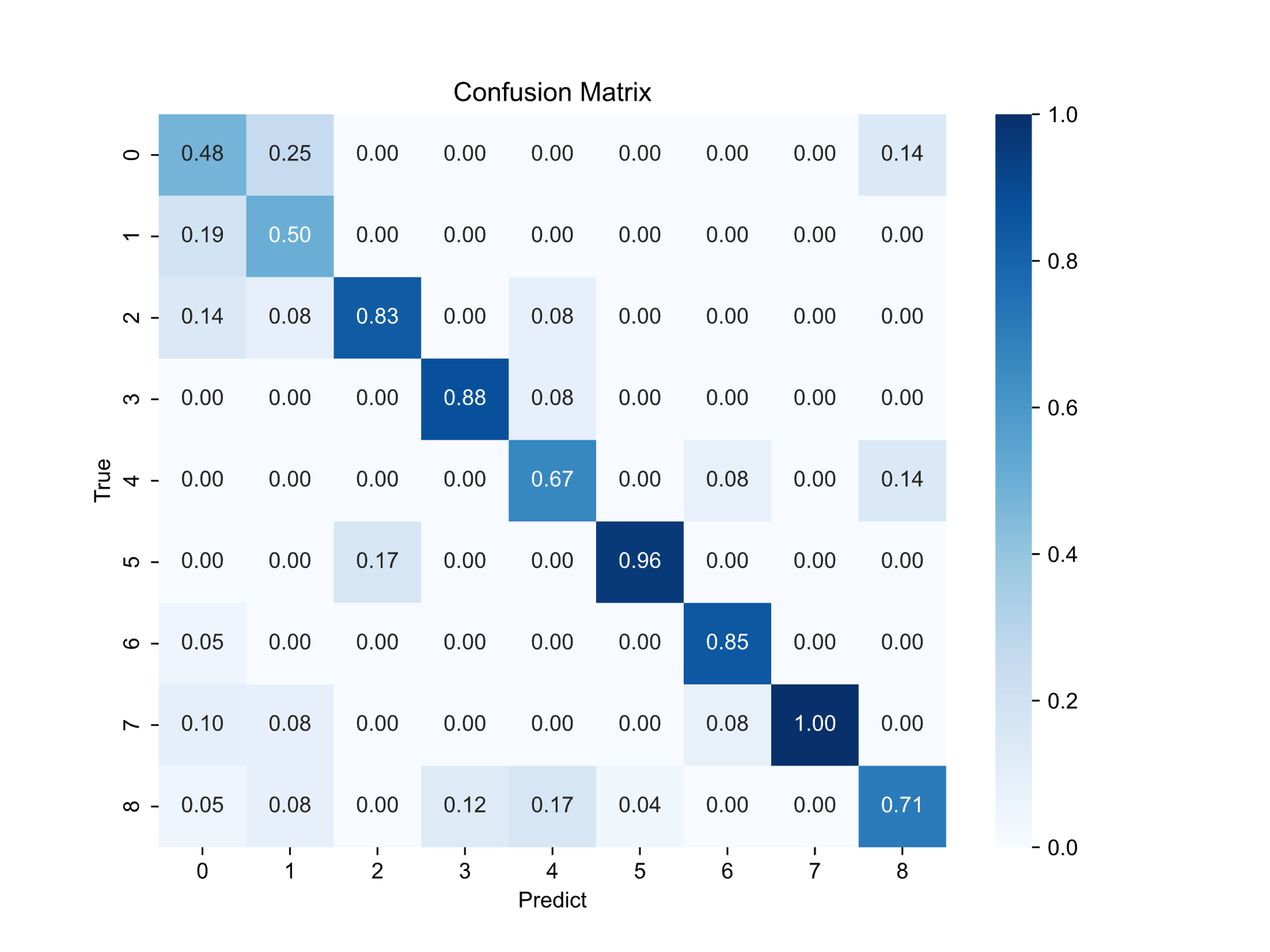** |
| **Q** | **R** |
| **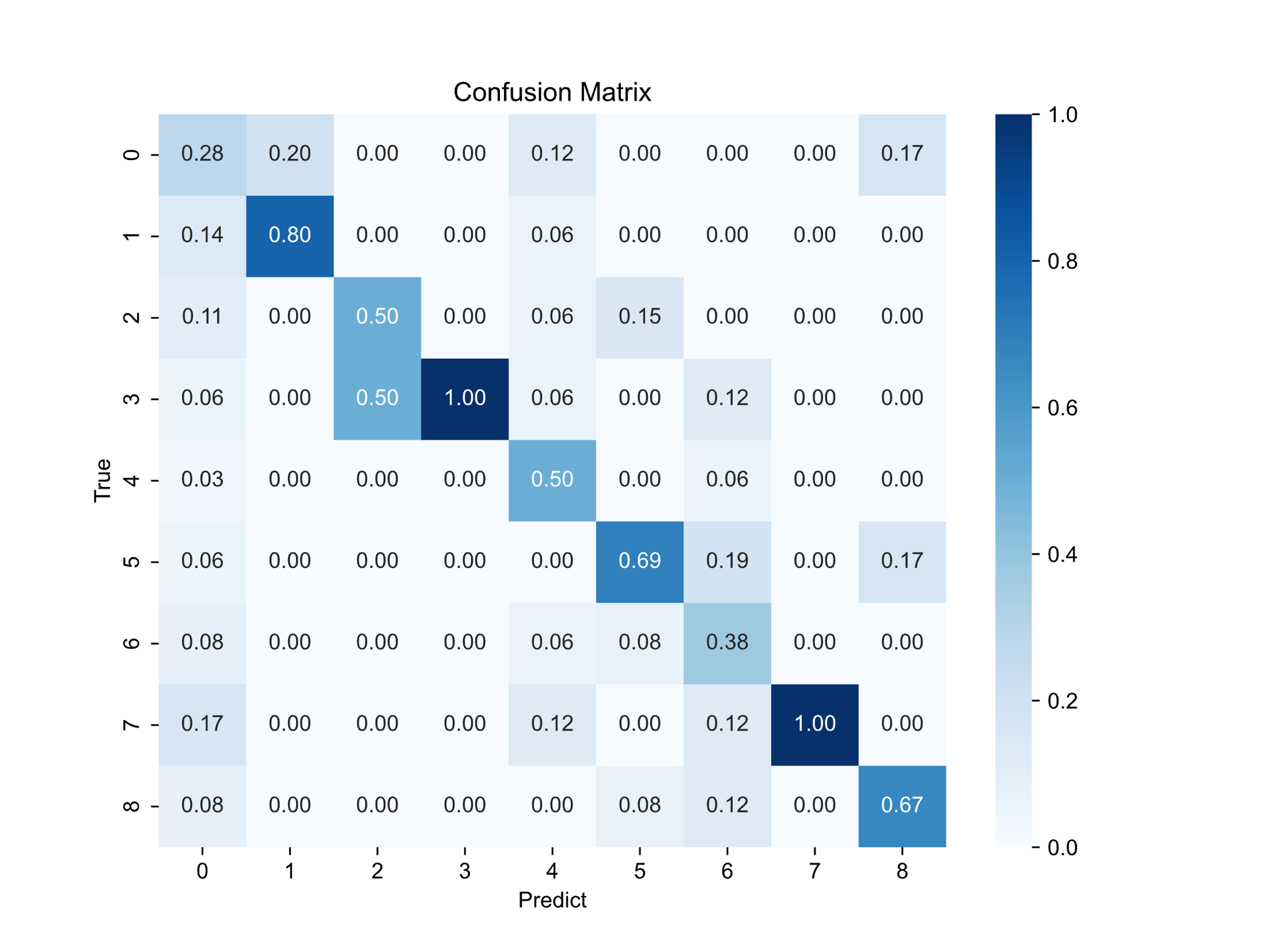** | **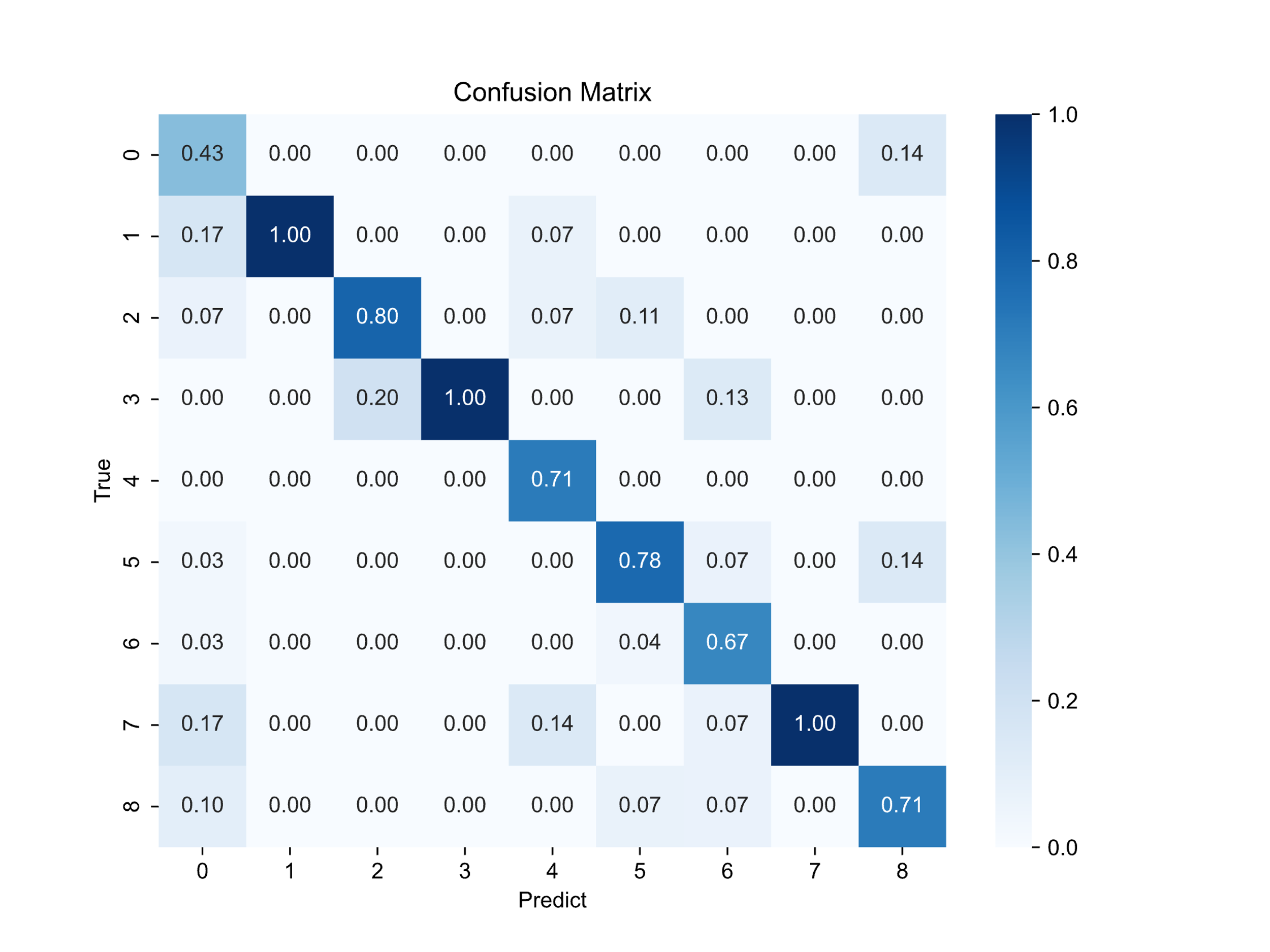** |

**Figure S1: Confusion matrix of the expert for top 3 and top 1 diagnosis.**

The left side represents the Top 1 diagnostic confusion matrices for Physicians 1-9; the right side represents the Top 3 diagnostic confusion matrices for Physicians 1-9. Class 0: Chronic obstructive pulmonary disease; Class 1: Bronchial asthma; Class 2: Bronchiectasis; Class 3: Airway stenosis; Class 4: Pulmonary hypertension; Class 5: Lung space-occupying lesions; Class 6: Pulmonary infectious diseases; Class 7: Pleural disease; Class 8: Interstitial lung disease.


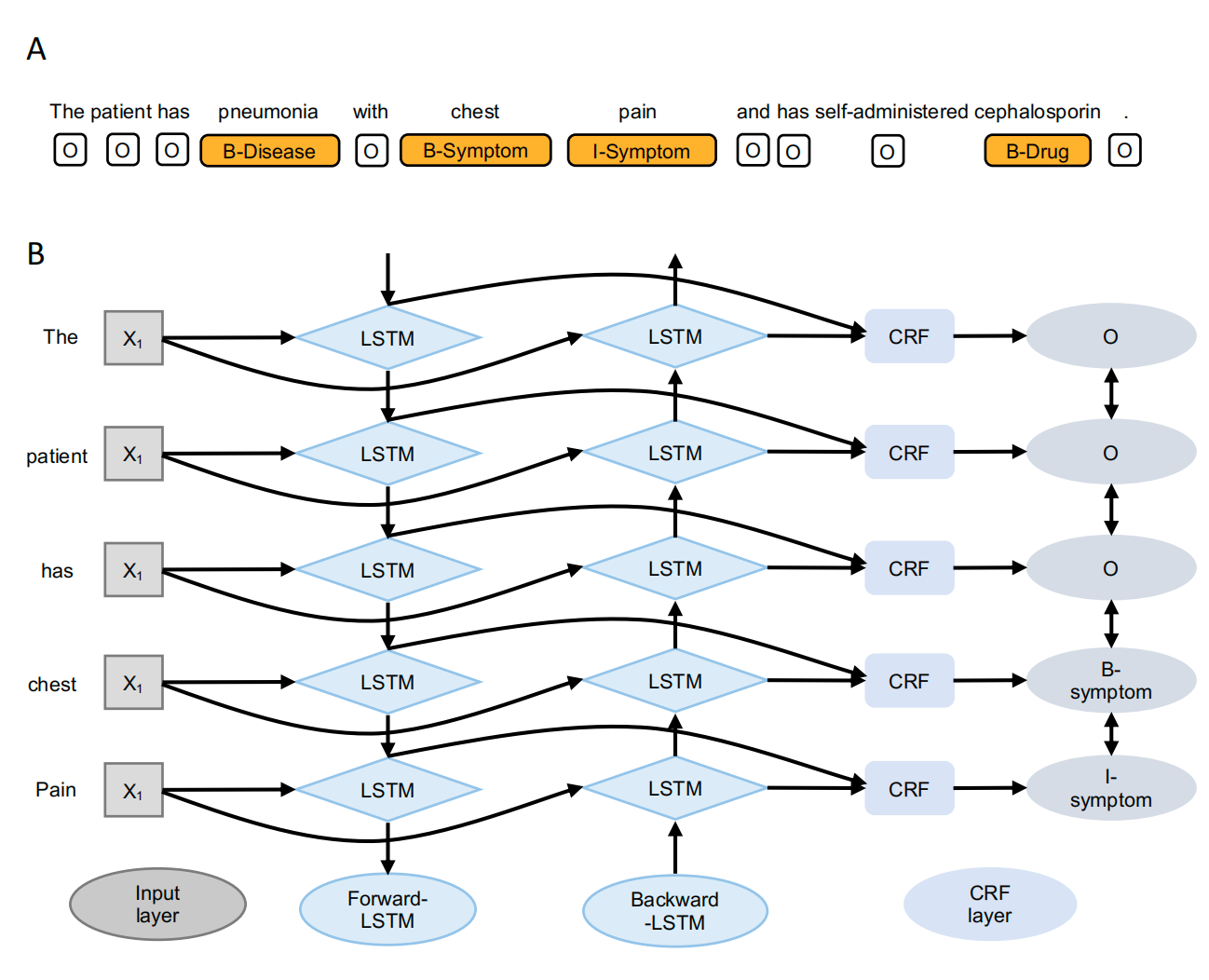


**Figure S2: Algorithm structure diagram**

A: "BIO" Rule-based annotation diagram, B-Tag: Represents the beginning of a named entity or a chunk of interest. It is assigned to the first token of the entity. I-Tag: Represents the continuation of a named entity. It is assigned to tokens that follow the initial token of the entity. O-Tag: Represents tokens that are outside any named entity or chunk of interest.; B: Phenotype recognition using Bidirectional Long Short-Term Memory (Bi-LSTM)- Conditional Random Field (CRF) deep learning model.


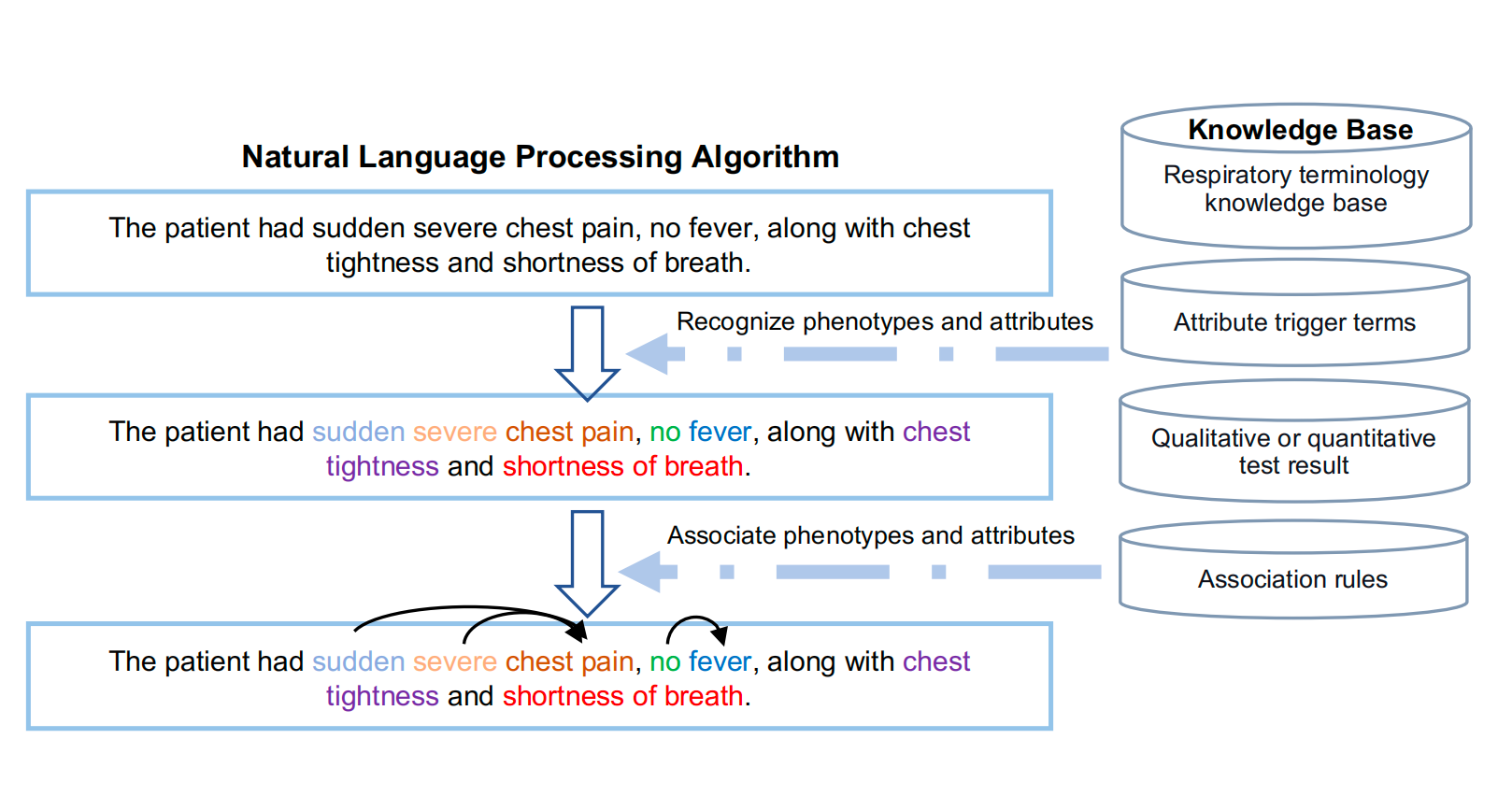


**Figure S3:** Phenotype and attribute correlation algorithm
